# The dynamics of AMPA receptors underlies the efficacy of ketamine in treatment resistant patients with depression

**DOI:** 10.1038/s41380-026-03510-w

**Published:** 2026-03-05

**Authors:** Waki Nakajima, Mai Hatano, Yohei Ohtani, Hideaki Tani, Taisuke Yatomi, Shohei Tsuchimoto, Yu Fujimoto, Tsuyoshi Eiro, Sadamitsu Ichijo, Kotaro Nakano, Tetsu Arisawa, Yuuki Takada, Kimito Kimura, Hiroki Abe, Akane Sano, Kie Nomoto-Takahashi, Kengo Yonezawa, Sota Tomiyama, Nobuhiro Nagai, Keisuke Kusudo, Shiori Honda, Sotaro Moriyama, Shinichiro Nakajima, Takashige Yamada, Yu Iwabuchi, Masahiro Jinzaki, Kimio Yoshimura, Shariful A. Syed, Sakiko Tsugawa, Hiroyuki Uchida, Takuya Takahashi

**Affiliations:** 1 Department of Physiology, Yokohama City University Graduate School of Medicine, Yokohama, 236-0004 Japan; 2 Department of Neuropsychiatry, Keio University School of Medicine, Tokyo, 160-8582 Japan; 3 Division of Neural Dynamics, Department of System Neuroscience, National Institute for Physiological Sciences, Okazaki, Aichi 444-8585 Japan; 4 Radioisotope Research Center, Yokohama City University Graduate School of Medicine, Yokohama, 236-0004 Japan; 5 Department of Psychiatry, Minami-Hanno Hospital, Saitama, 357-0042 Japan; 6 Department of Psychiatry and Behavioral Health, Renaissance School of Medicine at Stony Brook University, Stony Brook, NY USA; 7 Department of Anesthesiology, Keio University School of Medicine, Tokyo, 160-8582 Japan; 8 Department of Radiology, Keio University School of Medicine, Tokyo, 160-8582 Japan; 9 Department of Health Policy and Management, Keio University School of Medicine, Tokyo, 160-8582 Japan; 10 The International Research Center for Neurointelligence, Institutes for Advanced Study, University of Tokyo, Tokyo, 113-8654 Japan

**Keywords:** Predictive markers, Depression

## Abstract

Approximately 30% of patients with depression suffer from treatment-resistant depression (TRD). Ketamine has shown antidepressant efficacy for TRD. While glutamate α-amino-3-hydroxy-5-methyl-4-isoxazolepropionic acid receptor (AMPAR) has been demonstrated to play crucial roles in the process of pharmacological action of ketamine in experimental animals, it remains elusive how ketamine exhibits its efficacy through changes in AMPAR dynamics in patients with TRD. In this study, using a positron emission tomography (PET) tracer, [^11^C]K-2, which depicts AMPAR density in the living human brain, we detected a negative correlation between AMPAR density and illness severity and differences in AMPAR distribution between patients with TRD and healthy participants. Furthermore, we detected brain areas where ketamine administration altered AMPAR density in significant correlations with ketamine-induced antidepressant effect in patients with TRD. AMPAR density alteration in these regions partially rescued AMPAR phenotype in the affected areas. Thus, AMPAR dynamics underlies the antidepressant effect of ketamine in patients with TRD.

## Introduction

Major depressive disorder (MDD) is highly prevalent and often associated with significant morbidity, mortality, and substantial societal costs [1–4]. Approximately 30% of patients with MDD fail to respond to drug therapy [4], which is referred to as treatment-resistant depression (TRD). Ketamine has consistently shown robust antidepressant efficacy for TRD for the past 20 years [5, 6]. Glutamate α-amino-3-hydroxy-5-methyl-4-isoxazole propionic acid receptor (AMPAR), which is a principal component of neurotransmission in the brain [7–14], has been reported to mediate the antidepressant efficacy of ketamine in experimental animals [15–18]. In contrast, there has been no report as to how ketamine affects the dynamics of AMPAR in patients with TRD. Given the significant gap in the brain mechanisms between experimental animals and humans, the elucidation of pharmacological mechanisms in human is crucial for the development of an upgraded version of ketamine to overcome the critical weakness of ketamine’s short duration of antidepressant effect [5, 19–25] (i.e., a few weeks) and concerns regarding its long-term safety.

Previous animal studies have detected several candidate regions where ketamine-induced AMPAR modulation may be particularly relevant [15, 17, 22, 26–29]. In rodent models of depression, the prefrontal cortex, nucleus accumbens and hippocampus, which exhibit dysfunction of AMPAR, show increased AMPAR expression following ketamine administration [15, 22, 26, 28]. Conversely, the lateral habenula, which regulates monoaminergic neurotransmission, exhibits hyperactivity in depression models [17, 27]. Notably, ketamine treatment suppresses this hyperactivity in the lateral habenula via AMPAR antagonism [17] and also reduces the insertion of calcium-permeable AMPARs (CP-AMPARs) in ventral tegmental area dopamine neurons [29], thereby normalizing aberrant excitatory transmission within reward circuits. In human, recent neuroimaging studies have implicated multiple brain regions in the pathophysiology and treatment response of depression. Lesion network mapping (LNM) studies have identified the left dorsolateral prefrontal cortex (DLPFC) as a central node of depressive circuitry [30, 31], while the precuneus, a key region of the default mode network (DMN), exhibits ketamine-induced connectivity changes whose direction remains inconsistent [32]. Moreover, ketamine has been shown to modulate occipital cortical activity, as evidenced by increased glucose metabolism [33], enhanced functional connectivity [34], and gamma-band activation associated with clinical response [35]. However, the neuronal mechanisms underlying these imaging findings remain unclear [36–38]. Drawing on these previous findings, the dynamics of AMPARs across multiple brain regions have been suggested to play a critical role in the improvement of depressive symptoms. Therefore, we planned whole-brain analyses to identify all the brain regions involved in ketamine’s AMPAR-mediated effects in humans.

We recently developed a positron emission tomography (PET) tracer, [^11^C]K-2, the first and only technology which enables us to visualize and quantify the density of AMPAR in the living human brain [39]. Previously we demonstrated significant positive correlations between the imaging values of [^11^C]K-2 and the protein amount of AMPAR at each volume of interest (VOI) obtained from surgically resected tissues from patients with mesial temporal lobe epilepsy [39]. In vitro binding assays revealed that [^11^C]K-2 binds to all AMPAR subunits (GluA1–4) with moderate to high affinity [39], indicating that [^11^C]K-2 detects all subunits of AMPARs, including CP-AMPARs lacking GluA2. Further, Logan graphical analysis (LGA) of time activity curves (TACs) using white matter as a reference exhibited linearity, exhibiting the reversible binding kinetics of [^11^C]K-2 [39]. Importantly, standardized uptake value ratios (SUVR) calculated using white matter as a reference showed a strong positive correlation with *BP*_ND_ derived from LGA, indicating that SUVR serves as a reliable surrogate index of AMPAR density [39]. The amide residue at the terminal of [^11^C]K-2 was rapidly hydrolyzed after the tracer injection and converted to [^11^C]K-2_OH_. While in vitro study exhibited [^11^C]K-2_OH_ does not penetrate cell membrane, it passes blood brain barrier by the paracellular transport [40]. Further, [^11^C]K-2_OH_ is not a substrate of major efflux transporter [40]. In combination with reversible binding kinetics in LGA, [^11^C]K-2 injection (thus [^11^C]K-2_OH_) depicts cell surface AMPARs which are physiologically important fractions [40]. Thus, [^11^C]K-2_OH_ delineates the images [39]. In patients with epilepsy, we detected the increased uptake of the tracer where the current dipoles were located with Magneto-Encephalo-Graphy (MEG) compared to the other side [41]. Further, we found that local accumulation of AMPAR determined the amplitude of epileptic **γ** wave detected by electroencephalography (EEG) using [^11^C]K-2 [42]. We also characterized the specific distribution of AMPAR in patients with major psychiatric disorders (schizophrenia, bipolar disorder, depression, and autism spectrum disorder (ASD)) [43]. In this study, SUVR calculated using whole brain as a reference showed good correspondence with *BP*_ND_ derived from LGA [43]. In patients with depression, our previous study found a significant negative correlation between AMPAR density and symptom severity in the frontal to parietal lobes but detected no significant difference in AMPAR density compared to healthy participants [43]. These series of studies demonstrated the validity of [^11^C]K-2 to study neuropsychiatric disorders.

In this study, we visualized neuronal cell surface AMPA receptor expression in Japanese patients with TRD [44]. We detected brain regions where AMPAR density was negatively correlated with the depressive symptomatology score in patients with TRD. Further, we found that the density of AMPARs in some brain regions of patients with TRD was altered compared with that in healthy participants. We detected brain regions where AMPAR alteration significantly correlated with ketamine’s antidepressant effect. Some of these regions were overlapped with the above-mentioned affected brain areas in patients with TRD in a direction to rescue the alteration of AMPAR in TRD. Interestingly, in reward-related brain regions including the habenula, a decrease in the cell surface expression of AMPAR was significantly correlated with ketamine-induced antidepressant effect. Thus, AMPAR dynamics can be a crucial pharmacological target of ketamine in patients with TRD.

## Patients and methods

### Ethics statement

This study comprised three clinical studies that were registered under the following IDs: jRCTs031210124, UMIN000025132, and jRCTs031200083. The main ketamine study (jRCTs031210124) was approved by the Certified Review Board of Keio in accordance with the Ethical guidelines for medical and health research involving human participants by the Japan Ministry of Health, Labour and Welfare and the Clinical Trials Act in Japan. For comparison of healthy participants, the data were used in the other two studies (UMIN000025132, jRCTs031200083) which approved by Yokohama City University Human Investigation Committee and Yokohama City University Certified Institutional Review Board following the same ethical guidelines and the Clinical Trials Act in Japan. These studies were conducted at Yokohama City University Hospital, Keio University Hospital, and Kyushu University Hospital between August 2016 and October 2023. The specific roles of each site were as follows: recruitment, informed consent, administration of study drugs, and clinical assessments for patients with TRD in the main study were all conducted at Keio University Hospital. In the main study, PET/MRI imaging was performed at Keio University Hospital and Yokohama City University Hospital. Imaging healthy participants in the other two studies were conducted at Yokohama City University Hospital and Kyushu University Hospital. All participants provided written informed consent after receiving detailed information about the protocol. The CONSORT diagram is provided in Supplemental Fig. 1–3. Data from these three studies were combined and analyzed retrospectively.

### Participants

#### Patients with treatment resistant depression

The inclusion criteria were as follows: patients who (a) were in- and outpatients 20–59 years of age; (b) met the Diagnostic and Statistical Manual of Mental Disorders Fifth Edition (DSM-5) criteria for MDD using the structured clinical interview for DSM-5 [45], the Research version (SCID-5-RV [46]); (c) had an inadequate response defined as < 50% subjective improvement to approved doses of at least two antidepressants in the current episode based on a visual analogue scale ranging from 0 (no improvement) to 100 (complete improvement) [47–49]; (d) had a total score of ≥ 22 on the Montgomery Åsberg Depression Rating Scale (MADRS) at screening; and (e) had sufficient decision-making capacity confirmed by the MacArthur Competence Tool for Clinical Research [50]. The exclusion criteria are provided in the Supplementary information. Baseline PET imaging data were analysed for 34 participants. Baseline demographic and clinical characteristics did not differ significantly between the ketamine and placebo groups (Table 1). The mean age for the entire sample was 41.4 ± 9.4 years, 11 (32.4%) participants were female, and the mean duration of illness was 11.6 ± 8.2 years. The mean baseline MADRS total score was 28.1 ± 7.6. Details of concomitant medications are provided in Supplemental Table 1.The changes in the MADRS total scores and % improvement of the MADRS scores were -9.1 ± 9.9, 30.7 ± 31.4% in the ketamine group, −2.7 ± 5.1, 8.4 ± 22.5% in the placebo group (double-blind period), and −11.1 ± 8.7, 40.1 ± 26.5% in the placebo group (open-label period), respectively (the detail in Supplemental Table 2, 3). These clinical assessments were performed by one of the trained investigators who was blind to the results of the PET and MRI imaging.

**Table 1:** Baseline demographic and clinical characteristics of participants.

	All participants with TRD (N = 34)	Ketamine (N = 17)	Placebo (N = 17)	Healthy participants (N = 49)	P value
Age, years	41.9 ± 9.4	39.9 ± 9.5	42.9 ± 9.1	42.1 ± 7.2	0.38
Female, n (%)	11 (32.4)	6 (35.2)	5 (29.4)	19 (38.8)	0.71
Duration of illness, years	11.6 ± 8.2	10.8 ± 8.2	12.1 ± 7.9		0.65
Number of failed antidepressant trials	3.8 ± 1.7	3.8 ± 1.7	3.9 ± 1.6		0.84
Baseline MADRS total score	28.1 ± 7.6	29.2 ± 7.9	26.9 ± 7.1		0.38
Concomitant benzodiazepine use, n (%)	24 (70.6)	12 (70.6)	12 (70.6)		1.00

Values are shown as mean ± SD or n (%). P values are based on two-sample t tests for continuous variables and chi-square tests for categorical variables between the ketamine and placebo groups.

*MADRS*, Montgomery Åsberg Depression Rating Scale ;*TRD*, treatment-resistant depression.

#### Healthy participants

In the first study (UMIN000025132) the inclusion criteria were: healthy male participants who were 30–79 years of age and did not fulfil any diagnostic criteria for psychiatric conditions according to the DSM-IV [51] using the SCID-I/DSM-IV [46], DSM-5 [45] or ICD-10 [52]. Among them, age-matched (i.e., 30–59 years) healthy participants were included. In the second study (jRCTs031200083), the selection criteria were the same as those in the first study, other than the age range (i.e., 20–49 years) and sex (i.e., both men and women were included). The exclusion criteria are provided in the Supplementary information. Fifty-four healthy participants were included. After inclusion, thorough assessments identified that four participants met criteria for social communication disorder according to DSM-5 (n = 2) or had cognitive impairment (n = 2), and they were therefore excluded from further analysis. Additionally, one participant was excluded due to incompatibility of spatial normalization of [^11^C]K-2 image. The demographic characteristics of the remaining 49 participants are as follows: age, 42.1 ± 7.2years, 30 men and 19 women; number of participants in each age group: 19, 26, and 4 in 30 s, 40 s, and 50 s, respectively.

### Study design

A detailed explanation of this trial is described elsewhere [44]. Briefly, enrolled participants were randomly allocated to either the ketamine or placebo group in a 1:1 ratio. Both all participants and clinical raters were blind to the allocation during the double-blind period. The investigator responsible for study drug preparation, the pharmacist in charge of dispensing, and the supervising anesthesiologist were unblinded, but they had no involvement in any clinical or imaging assessments throughout the trial. During the double-blind treatment period, the baseline assessments including PET scan and clinical assessment were performed before the first administration of the study drug. In the ketamine group, intravenous ketamine (0.5 mg/kg) was administered over 40 min twice a week for two weeks (i.e., four times in total), whereas in the placebo group intravenous placebo (0.9% sodium chloride for injection) was administered over 40 min twice a week for two weeks. This repeated-infusion design was chosen to investigate the neurobiological mechanisms of ketamine’s sustained antidepressant effects rather than the transient acute effects. Both post-treatment clinical and PET imaging assessments were performed at 3.2 ± 1.5 days after the fourth administration of the study drug on the same day. Participants assigned to the ketamine group were terminated from the study at that time, while those assigned to the placebo group were then offered the opportunity to receive open-label, intravenous ketamine treatment. During this open-label treatment period, the participants received the treatment with intravenous ketamine (0.5 mg/kg) over 40 min twice a week for two weeks and received clinical assessment before and after this course of treatment. Participants continued the same dose of psychotropic medications that they were receiving at screening in both arms throughout the study period (see Supplemental Table 1).

The CONSORT diagram illustrates the flow of the participants (Supplemental Fig. 1). A total of 34 participants were assigned to receive either ketamine (n = 17) or placebo (n = 17) during the double-blind period. Three discontinued participations during the double-blind phase due to their intention to withdraw (n = 2 in the ketamine group) or fatigue (n = 1 in the placebo group). Subsequently, 16 participants in the placebo group received ketamine in the open-label extension period. Consequently, baseline PET imaging data were analysed for 34 participants (n = 17 in the ketamine group and n = 17 in the placebo group). For the analysis of the association between ΔSUVR_30-50_ and % improvement of MADRS, the matched pre- and post-treatment PET imaging data were analysed for 31 participants (n = 15 in the ketamine group and n = 16 in the placebo group).

### PET and MRI analysis procedure

MRI images scanned at Yokohama City University Hospital and Kyusyu University Hospital were segmented into probability maps of grey matter, white matter, and cerebrospinal fluid using a unified framework for tissue segmentation [53]. In MRI images scanned at Tokyo University, multiple segmentation was conducted using T1- and T2-weighed MRI. PET images scanned at Keio University Hospital were processed with a 5.0 mm full-width at half-maximum (FWHM) Gaussian filter to match the resolution of raw PET images scanned at other sites.

PET image taken between 30–50 min after injection of [^11^C]K-2 were summed and normalized to whole brain radioactivity, creating standardized uptake value ratio (SUVR)_30-50_ images. SUV of whole brain didn’t change in pre- and post-treatment with ketamine (pre-treatment: 2.44 ± 0.45, post-treatment: 2.34 ± 0.45, *p* = 0.471 (paired *t*-test)). All SUVR_30-50_ images were co-registered to each individual’s MRI. A template for anatomical normalization was created using the high-dimensional nonlinear warping algorithm DARTEL. SUVR_30-50_ images were spatially normalized into MNI standard space using the template with statistical parametric mapping (SPM) 12. Further details on the methodology were described in our previous study [43]. An 8 mm FWHM Gaussian filter was applied to the spatially normalized PET images.

### Detection of overlap areas

The binary images were created from reduction and increase regions compared with age-matched healthy participants, regions that showed a negative correlation between SUVR_30-50_ and MADRS, and regions that showed a positive and negative correlation between ΔSUVR_30-50_ and % improvement in MADRS. We identified the overlapped areas showing ketamine induced increase or decrease SUVR_30-50_ at affected areas in patients with TRD.

### Statistical analysis

A group comparison of SUVR_30-50_ between TRD patients and age-matched healthy participants was performed using two-sample *t*-test. Comparison of SUVR_30-50_ between before and after treatment with ketamine was using paired *t*-test. For the analysis of the association between ΔSUVR_30-50_ and % improvement in MADRS, we generated ΔSUVR_30-50_ images by subtracting pre-treatment from post-treatment SUVR_30-50_ images. MADRS scores improvement were calculated as: - (MADRS post-treatment – MADRS pre-treatment) / MADRS pre-treatment × 100%. Associations between SUVR_30-50_ and MADRS scores, ΔSUVR_30-50_ and MADRS improvement, and pre-treatment SUVR_30-50_ and MADRS improvement were assessed using multiple regression design implemented in SPM12. Covariates (age, sex) were included in the models for the analyses between SUVR_30-50_ and MADRS scores, and between pre-treatment SUVR_30-50_ and MADRS improvement, and group comparison, but not for the analysis between ΔSUVR_30-50_ and MADRS improvement, as the subtraction process minimizes the influence of these covariates.

To further assess potential confounding effects, we performed additional regression analyses between SUVR_30-50_ and each of the demographic and clinical variables (age, sex, illness duration, benzodiazepine use, and number of failed antidepressant trials). These variables were then included as covariates in sensitivity analyses to confirm the robustness of our primary results.

Statistical significance was set at *p* < 0.05 (peak-level uncorrected), false discovery rate (FDR) was corrected at *p* < 0.05 (cluster-level) for multiple comparisons across all in-mask voxels. To minimize false-positives, analyses were restricted to grey matter regions exceeding 10% probability based on standard tissue probability maps in SPM12. Additional correlation analysis and scatter plot were generated using GraphPad Prism 9 (Graph Pad Software, Massachusetts, USA). The assumption of homogeneity of variance for the group comparison was confirmed using Levene’s test. All data fulfil the normality assumption.

## Results

### The profiles of AMPAR in patients with TRD

**Fig. 1:**
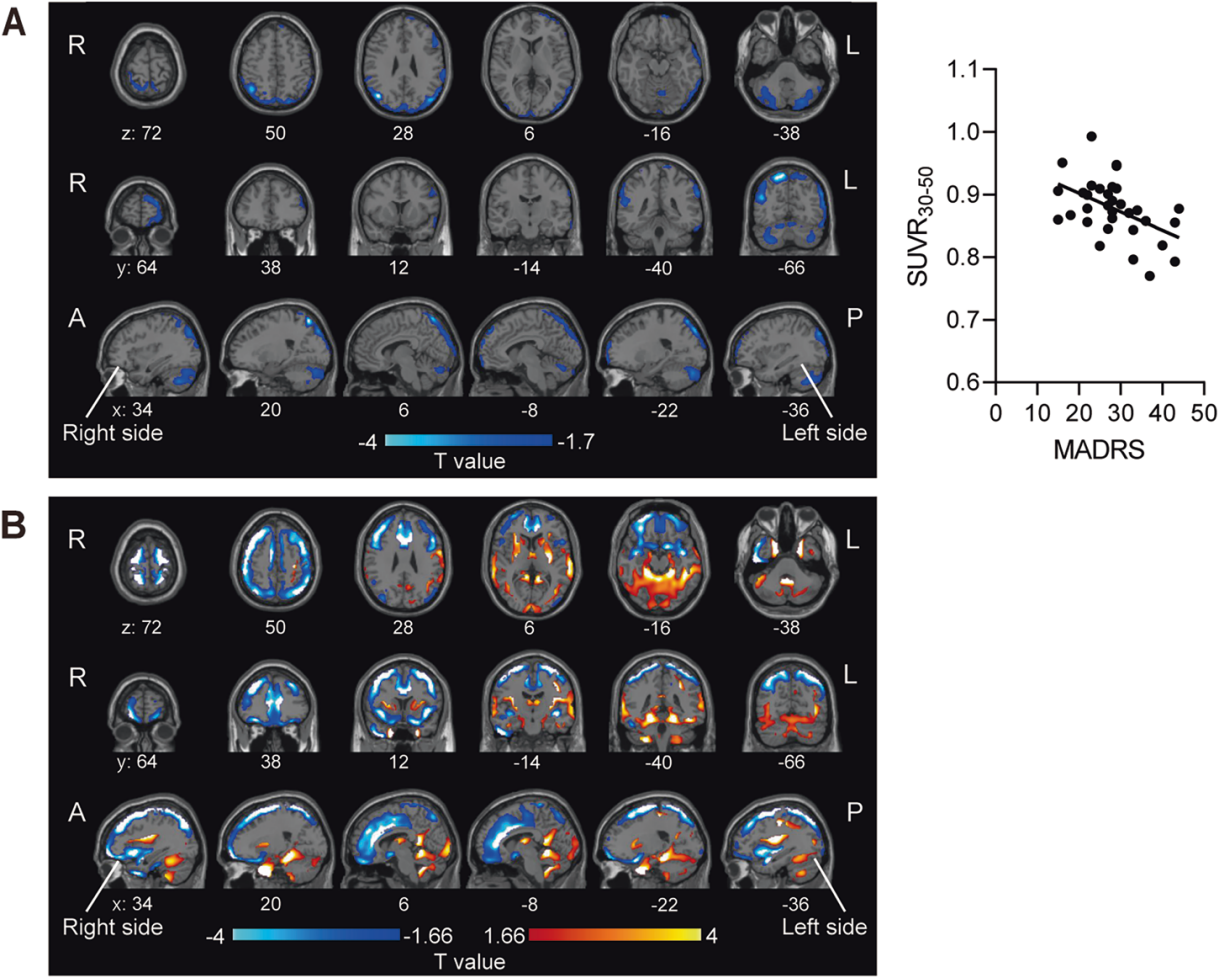
Characterization of AMPAR distribution in patients with TRD. **A**, Brain regions showing a significant negative correlation between SUVR_30-50_ and the MADRS scores in patients with TRD (*p* < 0.05, *t* < −1.7, one-tailed, FDRc). Significant clusters displayed on an axial, coronal, sagittal slices (Left), and scatter plot between averaged SUVR_30-50_ in significant cluster and the MADRS scores (Right, two-tailed Pearson correlation analysis: correlation coefficient = −0.4856). **B**, Relative reduction (blue) and increase (red) of SUVR_30-50_ in patients with TRD compared to healthy participants (*p* < 0.05, increase of SUVR_30-50_ : *t* > 1.66, reduction of SUVR_30-50_ : *t* < −1.66, one-tailed, FDRc). Significant clusters displayed on axial, coronal, and sagittal slices. **A** and **B** were adjusted for covariates (age and sex). The (x, y, z) value represents MNI coordinates of represented sections. FDRc; false discovery rate correction, R; Right, L; Left, A; Anterior, P; Posterior.

We have recently reported the phenotypes of the distribution of cell surface AMPAR in patients with depression that mainly consisted of patients with non-TRD using PET scanning with [^11^C]K-2 [43]. In the current study, we focused on patients with TRD and performed PET scanning on them. We performed a regression analysis between standardized uptake value ratio (SUVR) using the whole brain as a reference during 30- to 50- minutes after [^11^C]K-2 injection (SUVR_30-50_) and illness severity using statistical parametric mapping (SPM). We found a negative correlation between SUVR_30-50_ and the Montgomery Asberg Depression Rating Scale (MADRS) total score (higher values represent greater illness severity of depression) in the cortical areas including the left frontal, temporal, parietal, occipital lobes, and cerebellum (Fig. 1A, Supplemental Fig. 4A and Supplemental Table 4), indicating that alteration in AMPAR density within these regions may contribute to depressive states (“state” regions). Most affected regions spanning frontal to parietal cortex were similar to those detected in our previous study [43]. The occipital lobe and the cerebellum were newly identified regions specific to patients with TRD (Fig. 1A, Supplemental Fig. 4A and Supplemental Table 4). Next, we compared SUVR_30-50_ between patients with TRD and the healthy participants. We found that the decrease in SUVR_30-50_ in TRD in the anterior insula, anterior to middle cingulate cortex, frontal, parietal, occipital lobes, and the increase in the cerebellum, temporal lobe, thalamus, posterior insula, and basal ganglia compared to the healthy participants (Fig. 1B, Supplemental Fig. 4C and Supplemental Table 5). These regions were uniquely detected in patients with TRD, but not in patients with non-TRD [43].

To assess the influence of demographic factors, we examined associations between SUVR_30-50_ and age or sex within the TRD group. We found significant negative correlations between SUVR_30-50_ and age (Supplemental Fig. 5A and Supplemental Table 6), and significant increases and decreases in AMPAR density in male patients compared with female patients (Supplemental Fig. 5B and Supplemental Table 7). Given these findings, age and sex were included as covariates in the above analyses. Additionally, within the TRD group, neither illness duration, number of failed antidepressant trials, nor benzodiazepine use showed significant correlations with baseline AMPAR density (all *p* > 0.05, uncorrected). Sensitivity analyses including these variables as additional covariates in the within-group models yielded essentially unchanged results, confirming the robustness of our primary findings (Supplemental Fig. 4B).

### Ketamine alters cell surface presentation of AMPAR

**Fig. 2:**
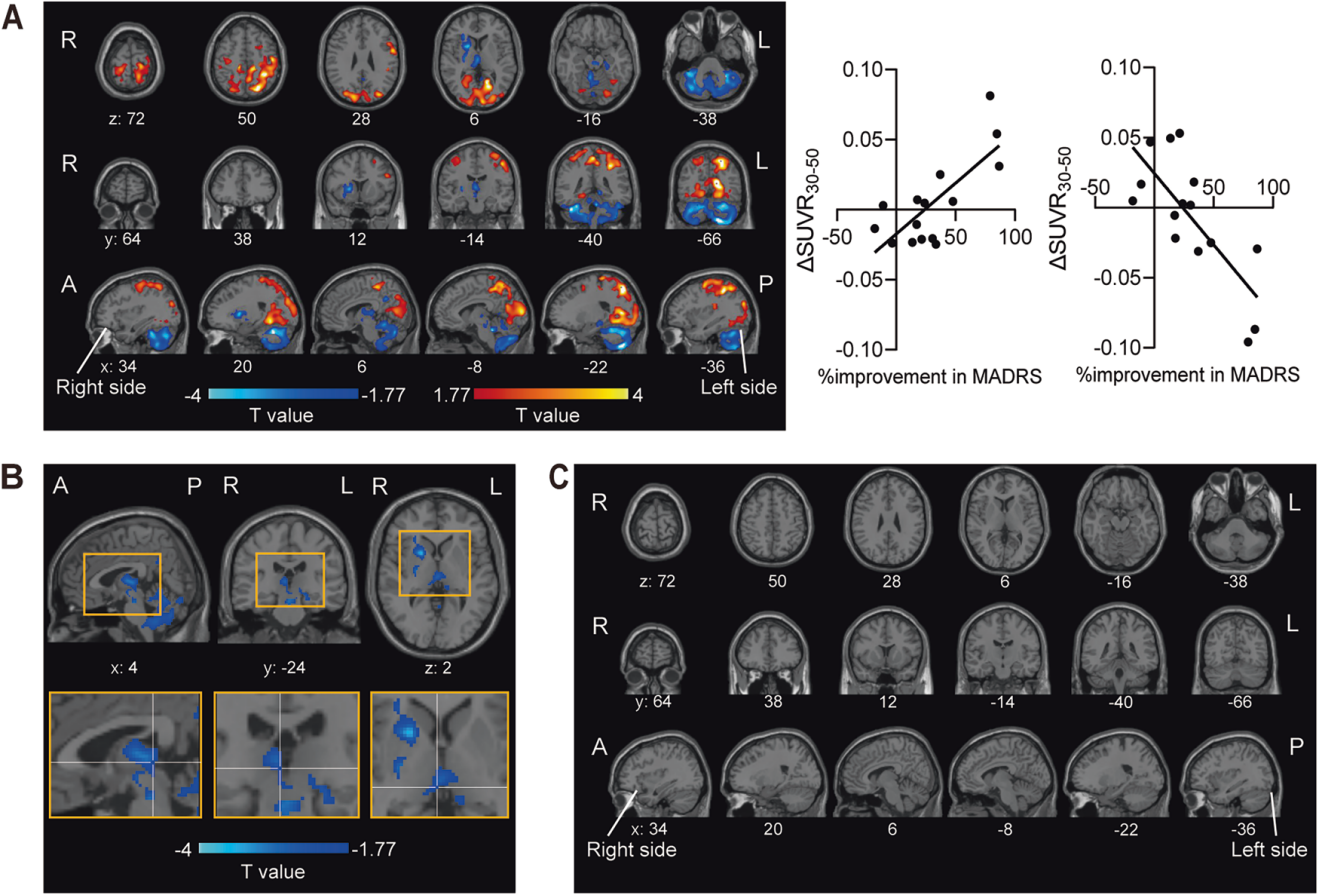
Changes in cell surface AMPAR associated with symptom improvement induced by ketamine administration. **A**, Brain regions showing a significant positive (red) and negative (blue) correlation between ΔSUVR_30-50_ and % improvement in MADRS in patients with TRD in the ketamine group (*p* < 0.05, positive correlation: *t* > 1.77, negative correlation: *t* < −1.77, one-tailed, FDRc). Significant clusters displayed on an axial, coronal, sagittal slices (Left), and scatter plot between averaged ΔSUVR_30-50_ in significant cluster and % improvement in MADRS (Right, two-tailed Pearson correlation analysis: positive correlation; correlation coefficient = 0.7536, negative correlation; correlation coefficient = −0.7536). This analysis was not adjusted for covariates. **B**, A significant negative (blue) correlation map between ΔSUVR_30-50_ and % improvement in MADRS in patients with TRD in the ketamine group was overlaid in the habenula. Significant clusters displayed on the MNI coordinate of the habenula (x = 4, y = −24 and z = 2, Top) and zoomed view of the habenula (The center of crossing line shows the habenula, Bottom). **C**, No significant correlation between ΔSUVR_30-50_ and % improvement in MADRS in patients with TRD in the placebo group. The (x, y, z) value represents MNI coordinates of represented sections. FDRc; false discovery rate correction, R; Right, L; Left, A; Anterior, P; Posterior.

Next, we investigated how ketamine administration alters the dynamics of AMPAR in patients with TRD. First, we compared the average SUVR_30-50_ images of the first scan (before the treatment with ketamine) to those of the second scan (after the treatment with ketamine) and there was no significant difference between them using a paired *t*-test with SPM. Next, we performed a regression analysis between ΔSUVR_30-50_ and improvement in depressive symptoms. We found the significant positive correlation between ΔSUVR_30-50_ and % improvement in MADRS in some brain areas such as the parietal lobe, occipital lobe, middle cingulate cortex, and a part of left frontal lobe in the ketamine group (Fig. 2A, Supplemental Fig. 6A and Supplemental Table 8). Interestingly, we found brain areas such as the cerebellum, thalamus, left parahippocampal gyrus, right basal ganglia showed a significant negative correlation between ΔSUVR_30-50_ and % improvement in MADRS in the ketamine group (Fig. 2A, Supplemental Fig. 6A and Supplemental Table 8). Notably, in the habenula, there existed a negative correlation between ΔSUVR_30-50_ and % improvement in MADRS (Fig. 2B). This suggested that ketamine-induced reduction in cell surface AMPAR in the habenula resulted in the antidepressant effect of ketamine. In contrast, there were no significant correlations in the placebo group (Fig. 2C). Thus, the effect of ketamine on AMPAR dynamics, which relieves depressive symptoms of patients with TRD, could depend on the brain regions.

**Fig. 3:**
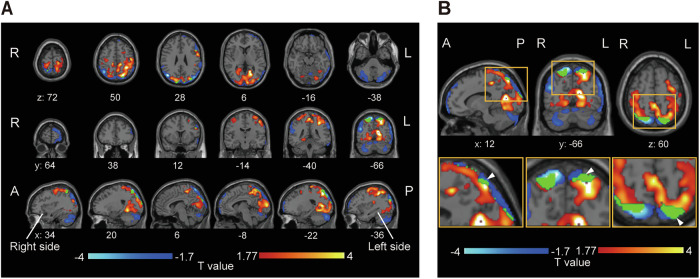
Overlapping regions where changes in AMPAR density correlate with clinical response to ketamine and regions where AMPAR density is altered in association with symptoms. **A**, Brain regions showing a significant negative correlation between SUVR_30-50_ and the MADRS scores in patients with TRD (blue) (*p* < 0.05, *t* < −1.7, one-tailed, FDRc, adjusted covariate (sex and age)) and a significant positive correlation between ΔSUVR_30-50_ and % improvement of MADRS in patients with TRD in the ketamine group (red) (*p* < 0.05, *t* > 1.77, one-tailed, FDRc, no adjusted covariate). Green region shows where the two regions overlap. Significant clusters and overlapping regions displayed on an axial, coronal, sagittal slices. **B**, Overlapping regions included the precuneus and the superior parietal cortex (x = 12, y = −66 and z = 60, Top) and zoomed view (arrow heads show the precuneus and the superior parietal cortex, Bottom). The (x, y, z) value represents MNI coordinates of represented sections. FDRc; false discovery rate correction, R; Right, L; Left, A; Anterior, P; Posterior.

As described above, we detected brain regions such as the parietal cortex and the occipital cortex that exhibited a negative correlation between SUVR_30-50_ and the MADRS scores in patients with TRD (Fig. 1A and Supplemental Fig. 4A). These regions were partially overlapped with brain areas, such as the parietal, occipital lobes, and a part of the frontal lobe that exhibited positive correlations between ketamine administration-induced increase in ΔSUVR_30-50_ and % improvement in MADRS (Fig. 3, Supplemental Fig. 7A and Supplemental Table 9).

**Fig. 4:**
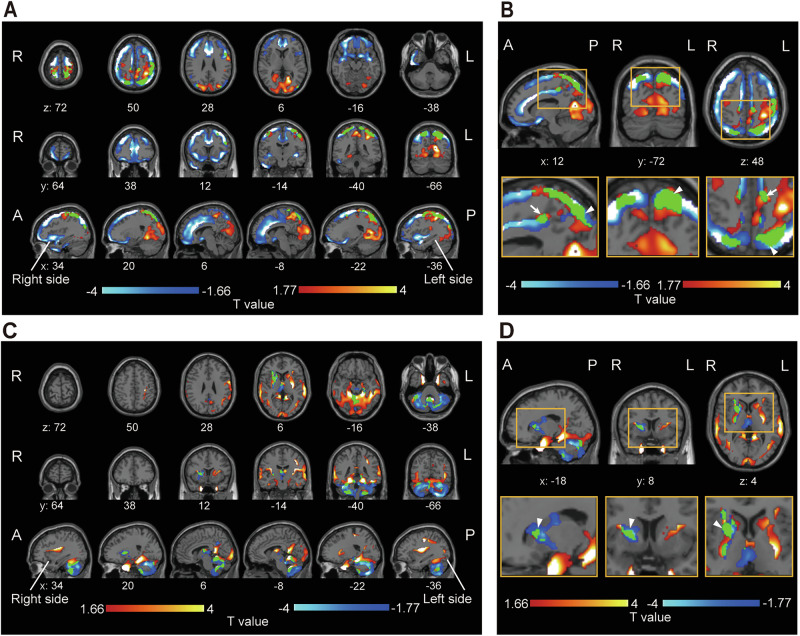
Overlapping regions where changes in AMPAR density correlate with clinical response to ketamine and regions where AMPAR density is different compared to healthy participants. **A**, Brain regions showing a significant relative reduction of SUVR_30-50_ in patients with TRD compared to healthy participants (blue) (*p* < 0.05, *t* < −1.66, one-tailed, FDRc, adjusted covariate (age, sex)) and a significant positive correlation between ΔSUVR_30-50_ and % improvement of MADRS in patients with TRD in the ketamine group (red) (*p* < 0.05, *t* > 1.77, one-tailed, FDRc, no adjusted covariate). Green region shows where the two regions overlap. Significant clusters and overlapping regions displayed on an axial, coronal, sagittal slices. **B**, Overlapping regions included the precuneus, the superior parietal cortex and the middle cingulate cortex (x = 12, y = −72 and z = 48, Top) and zoomed view (arrow heads show the precuneus and the superior parietal cortex, arrows show the middle cingulate cortex, Bottom). **C**, Brain regions showing a significant relative increase of SUVR_30-50_ in patients with TRD compared to healthy participants (red) (*p* < 0.05, *t* > 1.66, one-tailed, FDRc, adjusted covariate (age and sex)) and a significant negative correlation between ΔSUVR_30-50_ and % improvement of MADRS in patients with TRD in the ketamine group (blue) (*p* < 0.05, *t* < −1.77, one-tailed, FDRc, no adjusted covariate). Green region shows where the two regions overlap. Significant clusters and overlapping regions displayed on an axial, coronal, sagittal slices. **D**, Overlapping regions included the putamen and the pallidum (x = -18, y = 8 and z = 4, Top) and zoomed view (arrow heads show putamen and the pallidum, Bottom). The (x, y, z) value represents MNI coordinates of represented sections. FDRc; false discovery rate correction, R; Right, L; Left, A; Anterior, P; Posterior.

Next, we examined the effect of ketamine administration on the cell surface density of AMPAR in brain regions where we detected the difference between patients with TRD and healthy participants. We detected brain regions such as the parietal, occipital lobes, middle cingulate cortex, and a part of the left frontal lobe that exhibited reduced SUVR_30-50_ in patients with TRD compared with healthy participants and presented with a positive significant correlation between ΔSUVR_30-50_ and % improvement in MADRS by the ketamine administration (Fig. 4A, B, Supplemental Fig. 8A and Supplemental Table 10). In addition, we also found brain regions such as the cerebellum, right basal ganglia which exhibited increased cell surface AMPAR density in patients with TRD compared with healthy participants and showed a negative significant correlation between ΔSUVR_30-50_ and % improvement in MADRS by the ketamine administration (Fig. 4C, D, Supplemental Fig. 8C and Supplemental Table 10). Additionally, sensitivity analysis was performed including age, sex, illness duration, benzodiazepine use and the number of failed medication trials as covariates. While the overall pattern of results remained essentially unchanged (Supplemental Fig. 6B, 7B, 8B, 8D), we observed some differences: negative correlations between ketamine-induced improvement in depressive symptoms and changes in AMPAR density emerged in the bilateral basal ganglia (Supplemental Fig. 6B, 8D). These results are consistent with the concept that ketamine regulates the dynamics of AMPAR trafficking toward an antidepressant direction in patients with TRD.

### Cell surface distribution of AMPAR predicts a response to ketamine

**Fig. 5:**
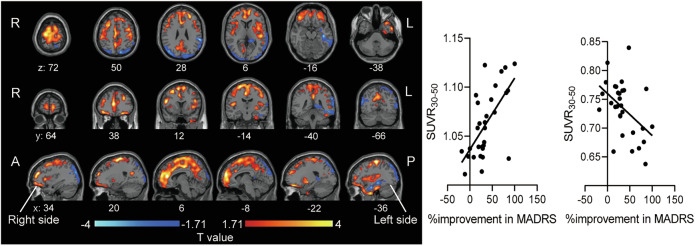
Brain regions where AMPAR distribution predicts ketamine response in patients with TRD. (Left) Brain regions showing a significant positive (red) and negative (blue) correlation between SUVR_30-50_ before ketamine administration and % improvement of MADRS (*p* < 0.05, positive correlation: *t* > 1.71, negative correlation: *t* < −1.71, one-tailed, FDRc, adjusted covariate (age and sex)). (Right) Scatter plot between averaged SUVR_30-50_ in significant cluster and % improvement of MADRS (two-tailed Pearson correlation analysis: positive correlation; correlation coefficient = 0.6361, negative correlation; correlation coefficient = −0.4609). The (x, y, z) value represents MNI coordinates of represented sections. FDRc; false discovery rate correction, R; Right, L; Left, A; Anterior, P; Posterior.

A significant positive correlation was found between SUVR_30-50_ before ketamine administration and % improvement in MADRS by the ketamine administration in brain areas such as the frontal, temporal, parietal, occipital lobes, insula, cingulate cortex, and basal ganglia (Fig. 5, Supplemental Fig. 9A and Supplemental Table 11). We also found brain regions such as the left temporal, occipital lobes and a part of the parietal lobe with significant negative correlations between SUVR_30-50_ before ketamine administration and % improvement in MADRS by the ketamine administration (Fig. 5, Supplemental Fig. 9A and Supplemental Table 11). Additionally, a sensitivity analysis was performed including age, sex, illness duration, benzodiazepine use and the number of failed medication trials as covariates. In this analysis, the positively correlated regions remained largely unchanged, whereas the negative correlations described in Fig. 5 did not reach significance (Supplemental Fig. 9B). Therefore, relative cell surface AMPAR density in some specific brain regions can determine the responsiveness to ketamine among patients with TRD.

## Discussion

In this study, we profiled AMPAR density on the cell surface in patients with TRD and how ketamine affected the dynamics of AMPAR in the living human brain. We detected cerebellar areas where we found a negative correlation between [^11^C]K-2 uptake and MADRS score in patients with TRD (Fig. 1A and Supplemental Figs. 4A, 4B). In our previous paper, we reported that there existed brain areas in the cerebellum which exhibited a negative correlation between [^11^C]K-2 uptake and Young Mania Rating Scale (YMRS) in patients with bipolar disorder [43]. In this report, these two areas were not overlapped so that there exist cerebellar area-specific roles on emotional processing.

In our previous study, which primarily included patients with non-TRD who had mild to moderate illness severity and showed a good response to conventional antidepressants, we detected no difference in [^11^C]K-2 uptake between patients with depression and healthy participants [43]. In contrast, we found remarkable differences between patients with TRD and healthy participants in this study (Fig. 1B and Supplemental Fig. 4C). Previously, we found regional differences in AMPA receptor density between psychiatric patients (e.g., bipolar disorder, schizophrenia and ASD) and healthy participants, and majority of these regions did not show significant correlations with symptom severity scores [43]. We hypothesized that these areas may be representative of alterations more closely related to the disease process itself, or a “trait region”. Similarly, we posit that patients with TRD share common trait-related regions across psychiatric disorders, which may lead them to present with distinct pathophysiology compared to non-TRD patients who experience a more normative treatment course. This supports the well-established notion that there are distinct biological processes subserving the treatment resistance trajectory. Part of these trait regions commonly exist across diseases (bipolar disorder, schizophrenia, ASD and TRD), while there exist disease specific trait regions. As was observed in the previous findings [43], we observed potential trait regions specific to TRD. Thus, while there exist common trait regions across psychiatric diseases, disease-specific trait regions also exist. It remains to be elucidated how these two types of traits emerge, what is the sequence of these events, and how these traits produce state regions.

We found that the habenula was included in the brain area that showed a significant correlation between the ketamine-induced antidepressant effect (% improvement of MADRS) and the reduction of cell surface AMPAR density (Fig. 2B). This is consistent with previous studies in experimental animals, which showed that ketamine administration in a depressive animal model reduced burst firing in the lateral habenula and that the changes in burst firing depended on AMPAR [17]. Interestingly, [^11^C]K-2 uptake in the habenula did not differ between TRD patients and healthy participants (Fig. 1B and Supplemental Fig. 4C), nor was it associated with baseline depressive severity (Fig. 1A and Supplemental Figs. 4A, 4B). A large body of evidence has been accumulated regarding the roles of the lateral habenula in depression in experimental animals [16–18], while it is still poorly elucidated how the lateral habenula contributes to the pathophysiology of depression in human. The lateral habenula has attracted wide attention in experiments with macaque monkey in which recordings of lateral habenula exhibited increased firing when animals fail to receive an expected reward or receive a cue predicting aversive stimuli [54]. While this is an acute response to negative rewards, repetitive negative rewards can cause depression. Animal models often employ such repetitive negative reinforcement to induce depression. However, these models differ substantially from TRD patients in chronicity, etiology, and genetic variability. Notwithstanding such a complicated situation, the decrease in cell surface AMPAR density in response to ketamine in the habenula in patients with TRD in the present study is consistent with the acute effect of ketamine on the lateral habenula of experimental animals [17]. This consistent finding is highly promising and critically important, considering that many biological outcomes differ between experimental animals and real-world human patients with depression in literature. Thus, biological events in the habenula can share the same mechanisms between experimental animals and patients with TRD at least in acute effects of ketamine.

As presented in our results, we identified brain regions showing either a positive or negative significant correlation between ketamine-induced improvement (% improvement in MADRS) and AMPAR density changes in patients with TRD (Fig. 2A, B and Supplemental Fig. 6). Brain areas with a positive correlation between ketamine-induced improvement and AMPAR density change were partially overlapped with brain regions where we detected a negative correlation between illness severity measured with MADRS and AMPAR density (Fig. 3 and Supplemental Fig. 7). These overlapping regions included the precuneus and the superior parietal cortex. Recent LNM studies have suggested that multiple brain regions, with the left DLPFC serving as a central node, are involved in the pathophysiology of depression [30, 31]. Specifically, brain areas such as the precuneus and the superior parietal cortex, which exhibit strong positive functional connectivity with the DLPFC, have been reported to show reduced activity and connectivity in depression, correlating with depressive symptoms. Our current findings in the precuneus and superior parietal cortex align with these LNM observations, reinforcing the idea that these regions play a pivotal role in depression, especially in the context of ketamine’s effect on AMPAR dynamics. The precuneus is a core region of the DMN, but its role, whether contributing to increased or decreased functional connectivity after ketamine treatment, remains unclear [32]. Given the positive correlation between AMPAR density and both short-range and long-range functional connectivity densities, AMPAR density may serve as a molecular determinant of functional connectivity [55]. Therefore, our findings suggest that ketamine may increase AMPAR density in association with depressive symptom improvement within specific DMN regions, potentially shifting them toward a normalized functional connectivity. Additionally, brain areas, such as the precuneus, the superior parietal cortex and the left middle cingulate cortex, where a positive correlation between ketamine-induced improvement and change in AMPAR density was also partially overlapped with brain regions with reduced AMPAR density compared with healthy participants (Fig. 4A, B and Supplemental Fig. 8A, 8B). Notably, the precuneus and middle cingulate cortex are components of para-cingulate network, which is associated with reward anticipation and working memory [56]. We also detected large brain regions in patients with TRD, including the putamen, the pallidum and the cerebellum, with increased AMPAR density than healthy participants. Some of these regions exhibited a negative correlation between ketamine-induced improvement and AMPAR density (Fig. 4C, D and Supplemental Fig. 8C, 8D). Among these, the pallidum plays a critical role in reward-based decision-making and provides input to the habenula. In particular, pallidal neurons projecting to the lateral habenula exhibit anti-reward characteristics [57]. Given that ketamine inhibits pallidal activity, it may suppress lateral habenula hyperactivity, thereby activating reward-related neural circuits. These results indicate that ketamine regulates AMPAR dynamics in a direction that rescues the phenotype of cell surface AMPAR in patients with TRD.

Our findings in the occipital cortex also warrant specific discussion. As shown in fMRI studies, patients with depression commonly report perceptual alterations, such as the “world appears colorless” or “foggy”, which are thought to reflect dysfunction in the visual network within the occipital lobe [58]. We observed that ketamine’s antidepressant effects were positively correlated with increases in AMPAR density in the occipital lobe (Fig. 2A and Supplemental Fig. 6). A MEG study similarly found that clinical response to ketamine in TRD participants was associated with gamma-band activation in the early visual cortex [35]. Moreover, prior imaging studies have shown that ketamine modulates occipital lobe function, as measured by increased glucose metabolism [33] and functional connectivity [34]. Taken together, these findings suggest that ketamine may restore visual network dysfunction in depression through AMPAR-mediated plasticity in the occipital lobe, offering a possible neurobiological mechanism for its antidepressant efficacy.

We also detected brain regions where there exists a positive or negative correlation between AMPAR density before the administration of ketamine and its subsequent antidepressant effect (Fig. 5 and Supplemental Fig. 9). [^11^C]K-2 may be a predictive tool for response to ketamine and may help guide treatment selection in patients with TRD.

Although significant associations were observed between AMPAR density and demographic factors such as age and sex, the absence of significant correlations with clinical variables including illness duration, number of failed antidepressant trials, and benzodiazepine use suggests that the observed alterations are not secondary to illness chronicity or treatment history. While most results remained essentially unchanged, we observed some differences: negative correlations between ketamine-induced improvement in depressive symptoms and changes in AMPAR density emerged in the bilateral basal ganglia (Supplemental Fig. 6B, 8D). In the analysis between pre-treatment SUVR_30-50_ and % improvement in MADRS by the ketamine administration, positive correlations remained, but no significant negative correlations were observed (Supplemental Fig. 9B). These findings suggest that the main conclusions of our study are robust to these potential confounders, although subtle regional differences may exist.

This study has several limitations. First, the sample size of the parent RCT [44] was determined based on the clinical score changes in TRD. Therefore, it may not be optimal for imaging analysis, limiting the interpretability of our findings. Further studies with a larger and more sex-balanced sample are warranted to confirm our results. Second, this study was conducted exclusively in a Japanese population, which may restrict the generalizability of the results to other ethnic or demographic groups. Third, our findings were analyzed within the range of MADRS improvement observed in the parent RCT [44], and variations in this range may influence the results. Thus, future studies should include patients with more severe baseline depression to investigate AMPAR dynamics across a broader range of clinical severity changes. Fourth, this study did not use an active placebo to avoid its potential confounding neurobiological effects. However, the adverse events associated with ketamine may have led to unblinding for the participants and raters. Although subjective expectations or allocation guesses were not assessed, such factors could have contributed to variability in symptoms through placebo or nocebo effects, thereby influencing symptom outcomes. Nevertheless, the robust correlation between changes in AMPAR density and clinical improvement observed only in the ketamine group suggests that [^11^C]K-2 PET signals can capture a biological association between AMPAR dynamics and the therapeutic effect of ketamine.

Overall, our findings demonstrate that AMPAR distribution is altered in patients with TRD and that ketamine partially normalizes these abnormalities in association with its antidepressant effects. These findings indicate that AMPAR dynamics underlies the antidepressant effect of ketamine in patients with TRD, highlighting AMPARs as a potential effector and biomarker of treatment response.

## Supplementary information

Supplementary Information

## Data Availability

As for one trial (jRCTs031210124), the trial protocol or the data that support the findings of this study are available from the corresponding author upon reasonable request. The Certified Review Board of Keio did not permit the deposit of raw data in a publicly accessible data archive or repository at the time of approval, as the procedure was not included in the protocol or informed consent document. Regarding the other two trials (UMIN000025132, jRCTs031200083), all requests for raw and analyzed data are promptly reviewed by the Yokohama City University Research Promotion Department to determine whether the request is subject to any intellectual property or confidentiality obligations and, further, inspected by the Institutional Review Board of Yokohama City University Hospital. Upon these approvals, derived data will be released via a material transfer agreement from the corresponding author.
