# Supplementary material for: The dynamics of AMPA receptors underlies the efficacy of ketamine in treatment resistant patients with depression
Source: Mol Psychiatry. 2026 Mar 5;31(7):3801–10. doi: 10.1038/s41380-026-03510-w (PMC13269144; doi:10.1038/s41380-026-03510-w)
Supplement: Supplementary file 1 — Supplementary Information [file 41380_2026_3510_MOESM1_ESM.docx]

**Supplementary information**

**Title: The dynamics of AMPA receptors underlies the efficacy of ketamine in treatment resistant patients with depression**

**Authors:** Waki Nakajima^1#^, Mai Hatano^1#^, Yohei Ohtani^2#^, Hideaki Tani^2^, Taisuke Yatomi^1,2^, Shohei Tsuchimoto^3^, Yu Fujimoto^1^, Tsuyoshi Eiro^1^, Sadamitsu Ichijo^1^, Kotaro Nakano^1^, Tetsu Arisawa^4^, Yuuki Takada^1^, Kimito Kimura^1^, Hiroki Abe^1^, Akane Sano^1^, Kie Nomoto-Takahashi^2^, Kengo Yonezawa^2^, Sota Tomiyama^2^, Nobuhiro Nagai^2,5^, Keisuke Kusudo^2^, Shiori Honda^2,6^, Sotaro Moriyama^2^, Shinichiro Nakajima^2^, Takashige Yamada^7^, Yu Iwabuchi^8^, Masahiro Jinzaki^8^, Kimio Yoshimura^9^, Shariful A. Syed^1^, Sakiko Tsugawa^1,2^, Hiroyuki Uchida^2^*, and Takuya Takahashi^1,10^*

**Affiliations:**

^1^ Department of Physiology, Yokohama City University Graduate School of Medicine, Yokohama, 236-0004, Japan.

^2^ Department of Neuropsychiatry, Keio University School of Medicine, Tokyo, 160-8582, Japan.

^3^ Division of Neural Dynamics, Department of System Neuroscience, National Institute for Physiological Sciences, Okazaki, Aichi 444-8585, Japan

^4^ Radioisotope Research Center, Yokohama City University Graduate School of Medicine, Yokohama, 236-0004, Japan

^5^ Department of Psychiatry, Minami-Hanno Hospital, Saitama, 357-0042, Japan

^6^ Department of Psychiatry and Behavioral Health, Renaissance School of Medicine at Stony Brook University, Stony Brook, NY, USA.

^7^ Department of Anesthesiology, Keio University School of Medicine, Tokyo, 160-8582, Japan

^8^ Department of Radiology, Keio University School of Medicine, Tokyo, 160-8582, Japan

^9^ Department of Health Policy and Management, Keio University School of Medicine, Tokyo, 160-8582, Japan

^10^ The International Research Center for Neurointelligence, Institutes for Advanced Study, University of Tokyo, Tokyo, 113-8654, Japan.

^#^These authors contributed equally to this work.

*Correspondence author: Hiroyuki Uchida (hiroyuki_uchida@keio.jp) and Takuya Takahashi (takahast@yokohama-cu.ac.jp)

**Supplementary Methods**

**Sample size**

Based on Singh et al.’s double-blind, randomized, placebo-controlled trial of repeated ketamine infusions (four times over two weeks) in U.S. patients with TRD, where MADRS score changes were −18.4 ± 12.0 (ketamine) and −5.7 ± 10.2 (placebo) with an effect size of 1.14, a sample of 14 patients per group was required to achieve 80% power (two-sided t-test, α = 0.05) ^1^. Allowing for a 14.7% (5 of 34) dropout rate, the target sample size was set at 17 per group (34 in total).

**Exclusion criteria**

*Patients with treatment resistant depression*. The inclusion criteria were provided in manuscript. The exclusion criteria were as follows: participants were excluded if they were pregnant, nursing, or planning to become pregnant; had a history of epilepsy; met substance abuse criteria within six months of the study; had a positive urine drug screen for illicit substances; had received either esketamine or ketamine; had a history of hypersensitivity to ketamine; had contraindications for MRI scanning; had significant neurological or general medical conditions; or had abnormal laboratory test values, specifically serum creatinine ≥ 1.5 mg/dL, AST ≥ 150 IU/L, or ALT ≥ 150 IU/L.

*Healthy participants.* In the first study (UMIN000025132) and the second study (jRCTs031200083), the inclusion criteria were provided in manuscript. Exclusion criteria were the same for the two studies: participants were excluded if they were pregnant, nursing, or planning to become pregnant; had a history of epilepsy; met substance abuse criteria within six months of the study; had a positive urine drug screen for illicit substances; had been treated with perampanel; had contraindications for MRI scanning; had significant neurological or general medical conditions; or had abnormal laboratory test values, specifically serum creatinine ≥ 1.5 mg/dL, AST ≥ 150 IU/L, or ALT ≥ 150 IU/L.

**Randomization and blinding**

Participants were randomly assigned to one of two treatment groups in a 1:1 ratio using a computer-generated randomization scheme, balanced by two or four randomly permuted blocks without stratification. The principal investigator (HU) and the designated investigator (HT) communicated the allocation via e-mail only to the investigator responsible for study drug preparation (T. Yatomi), the anesthesiologist supervising drug administration (T. Yamada), and the pharmacists in charge of dispensing. During the double-blind period, neither participants nor clinical assessors were informed of the allocation. Anyone who knew about the assignment was not involved in data entry for the case report form until final data lock.

**In vivo PET and MRI imaging.**

The participants were conducted MRI and PET scan with [^11^C]K-2, which was synthesized at each site according to GMP ordinance. Injected dose of [^11^C]K-2 were 376.4 ± 8.8 MBq (healthy participants), 371.8 ± 12.3 MBq (patients with TRD in pre-treatment scan), and 371.4 ± 6.7 MBq (patients with TRD in post-treatment scan). In previous study ^2^, we performed PET scan on all types of PET cameras using brain tumor (BT) phantom (Itoi Factory Inc.) ^3^ to validate reconstruction parameters. We could obtain the PET images which have the same resolution.

*Yokohama City University Hospital.* PET imaging was performed with a TOSHIBA Aquiduo scanner (TOSHIBA Medical) and a Celesteion PCA-9000A/2A (Canon medical). Healthy participants were PET-scanned by the Aquiduo, which provided an axial FOV of 240 mm and 80 contiguous 2.0 mm thick slices. After 4.7 s Computed Tomography (CT) scan for attenuation correction (AC), a 60 s injection of [^11^C]K-2 was given intravenously and a PET scan of 60 min was conducted in all studies. PET images were reconstructed by the following parameters: a 2D-OSEM using 4 iterations, 14 subsets, a 128 matrix, a zoom of 2.8 and a 5.0 mm Gaussian filter. Healthy participants and patients with TRD were PET-scanned by Celesteion provided an axial FOV of 240 mm and 96 contiguous 2.0 mm-thick slices. After 15.2 s CT scan, [^11^C]K-2 was administered intravenously and a PET scan was conducted. PET images were reconstructed by the following parameters: 3D-OSEM + TOF using 2 iterations, 20 subsets, a 128 matrix, a zoom of 1.0, and a 5.0-mm Gaussian filter. Each participant underwent MRI scan using a GE DISCOVERY MR750 (General Electric Medical Systems). High resolution 3D-T1-weighted images (T1WI) were acquired using the following parameters: voxel size = 0.9 × 0.9 × 0.9 mm, repetition time (TR) / time to echo (TE) = 7.0/3.1 ms, flip angle (FA) = 8°, FOV = 220 mm, Matrix = 256 × 256.

*Keio University Hospital.* Healthy participants and patients with TRD were PET-scanned by a Biograph mCT Flow (Siemens Medical Solutions), which provided an axial FOV of 300 mm, and 111 contiguous 2.0 mm thick slices. After 6.56 s CT scan, [^11^C]K-2 was administered intravenously and a PET scan was conducted. PET images were reconstructed by the following parameters: 3D-OSEM + TOF using 4 iterations, 24 subsets, a 200 matrix, a zoom of 2.0 and a 2.0 mm Gaussian filter. Each participant underwent an MRI scan on a MAGNETOM Prisma (SIEMENS Healthineers) at university of Tokyo. High resolution 3D-T1WI were acquired using 3D MPRAGE protocol (voxel size = 0.8 × 0.8 × 0.8 mm, TR/TE = 2400/2.22 ms, FA = 8°, FOV = 208 mm, Matrix = 300 × 320 mm^2^).

*Kyushu University Hospital.* PET imaging was performed with a Biograph mCT Flow (Siemens Medical Solutions) and a Biograph Vision (Siemens Medical Solutions). Healthy participants were PET-scanned by a Biograph mCT provided an axial FOV of 250 mm, and 165 contiguous 1.0 mm thick slices. After 9.38 s CT scan, [^11^C]K-2 was administered intravenously and a PET scan was conducted. PET images were reconstructed by the following parameters: a 3D-OSEM + TOF using 5 iterations, 21 subsets and a 5.0 mm Gaussian filter. Healthy participants were PET-scanned by a Biograph Vision provided an axial FOV of 357 mm, and 263 contiguous 1.0 mm thick slices. After 26.67 s CT scan, [^11^C]K-2 was administered intravenously and a PET scan was conducted. PET images were reconstructed by the following parameters: a 3D-OSEM + TOF using 8 iterations, 5 subsets and a 5.0 mm Gaussian filter. Each participant underwent an MRI scan using an Ingenia 3.0-T scanner (Phillips). High resolution 3D-T1WI were acquired using 3D MPRAGE protocol (voxel size = 1.2 × 1.0 × 1.0 mm, TR/TE = 6.8/3.1 ms, FA = 9°, FOV = 170 mm, Matrix = 256 × 256).

**References.**

1. Singh JB, Fedgchin M, Daly EJ, De Boer P, Cooper K, Lim P *et al.* A Double-Blind, Randomized, Placebo-Controlled, Dose-Frequency Study of Intravenous Ketamine in Patients With Treatment-Resistant Depression. *Am J Psychiatry* 2016; **173**(8)**:** 816-826.

2. Hatano M, Nakajima W, Tani H, Uchida H, Miyazaki T, Arisawa T *et al.* Characterization of patients with major psychiatric disorders with AMPA receptor positron emission tomography. *Molecular Psychiatry* 2025; **30**(5)**:** 1780-1790.

3. Standard PET imaging protocols and phantom test procedures and criteria: executive summary. 2017, Accessed Date Accessed 2017 Accessed.

**CONSORT diagram of trial no. jRCTs031210124**

**(Treatment-resistant depression)**


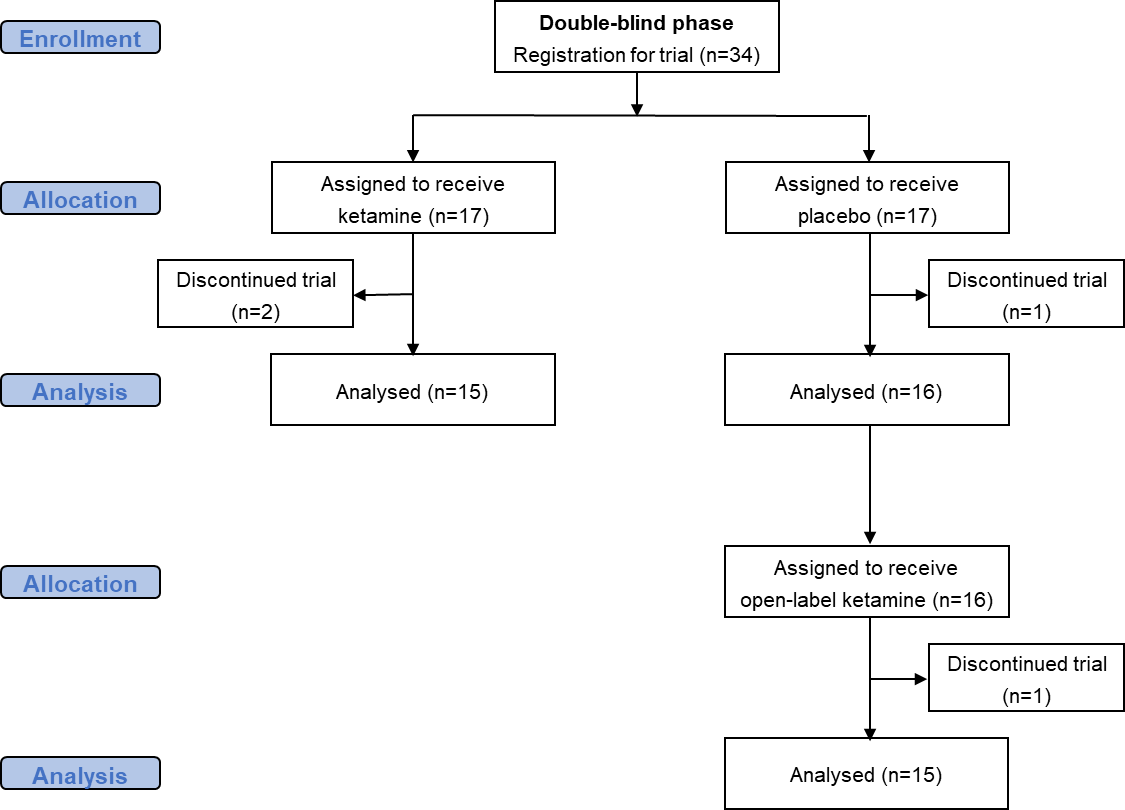


**Supplemental Fig. 1 CONSORT diagram of trial no. jRCTs031210124**

**CONSORT diagram of trial no. jRCTs031200083**

**(Healthy Participants)**

**
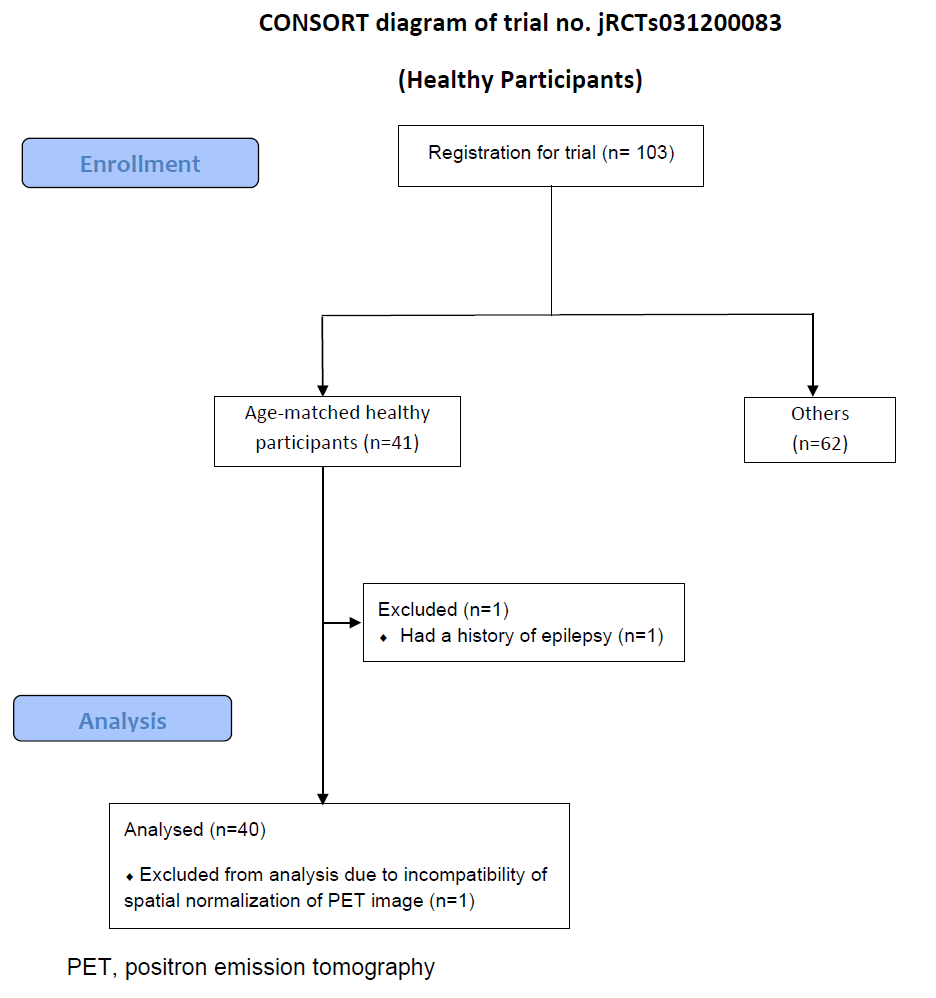
**

**Supplemental Fig. 2 CONSORT diagram of trial no. jRCTs031200083**

**CONSORT diagram of trial no. UMIN000025132**

**(Healthy Participants)**

**
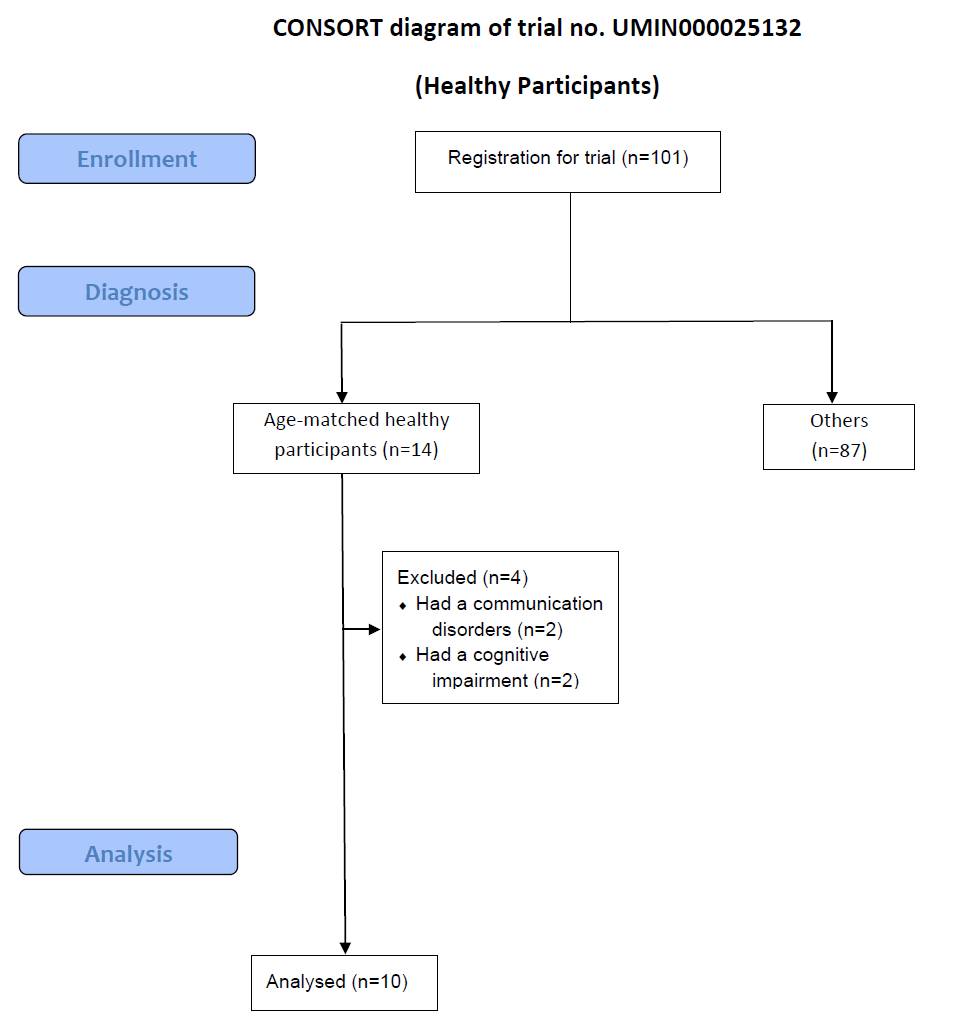
**

**Supplemental Fig. 3 CONSORT diagram of trial no. UMIN000025132**

**
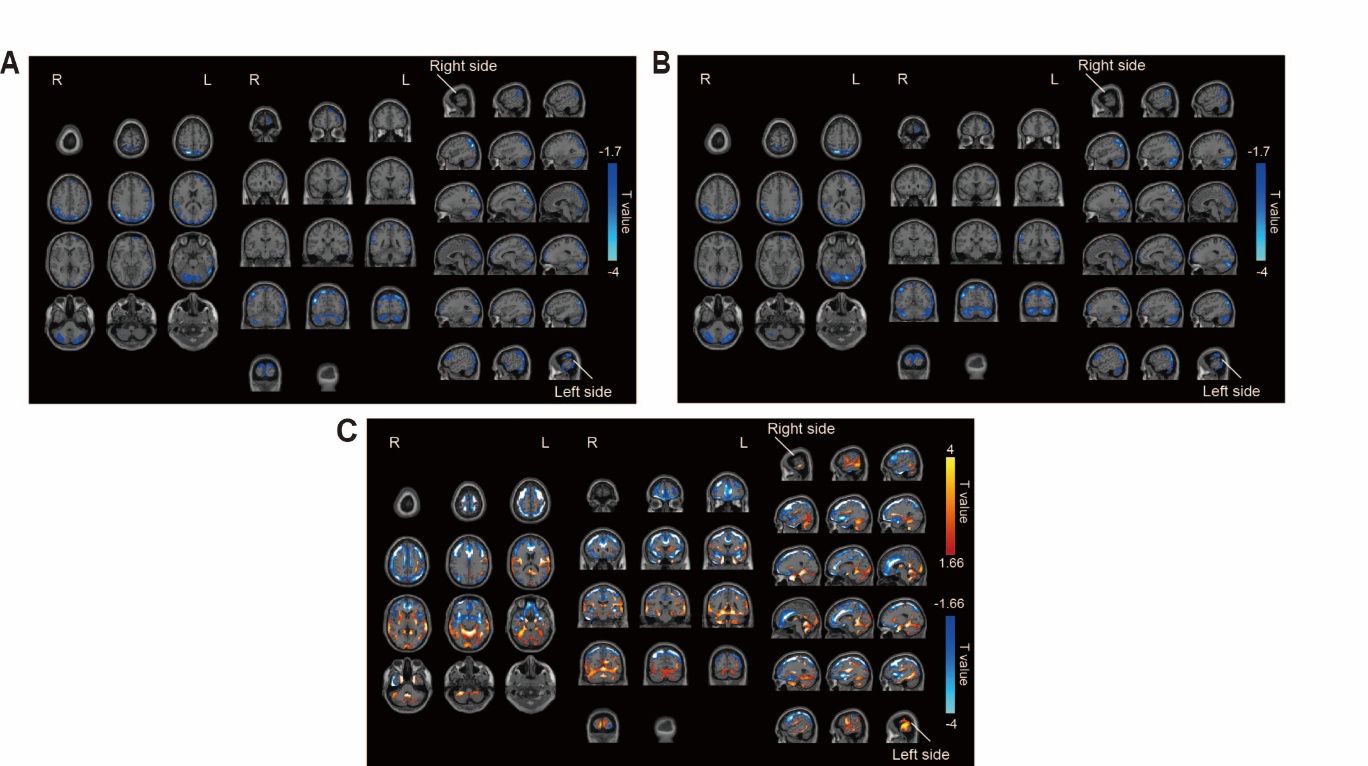
**

**Supplemental Fig. 4 Characterization of AMPAR distribution in patients with TRD (brain wide presentation)**

**A, B,** Brain region showing a significant negative correlation between SUVR_30-50_ and the MADRS scores in patients with TRD (*p* < 0.05, *t* < -1.7, one-tailed, FDRc). **C,** Relative reduction (blue) and increase (red) of SUVR_30-50_ in patients with TRD compared to healthy participants (*p* < 0.05, increase of SUVR_30-50_ : *t* > 1.66, reduction of SUVR_30-50_ : *t* < -1.66, one-tailed, FDRc). Significant clusters displayed on axial, coronal, sagittal slices. These analyses were adjusted for covariates (age and sex (**A** and **C**); and age, sex, illness duration, number of failed medication trials and usage of benzodiazepines (**B**)). FDRc; false discovery rate correction.

**
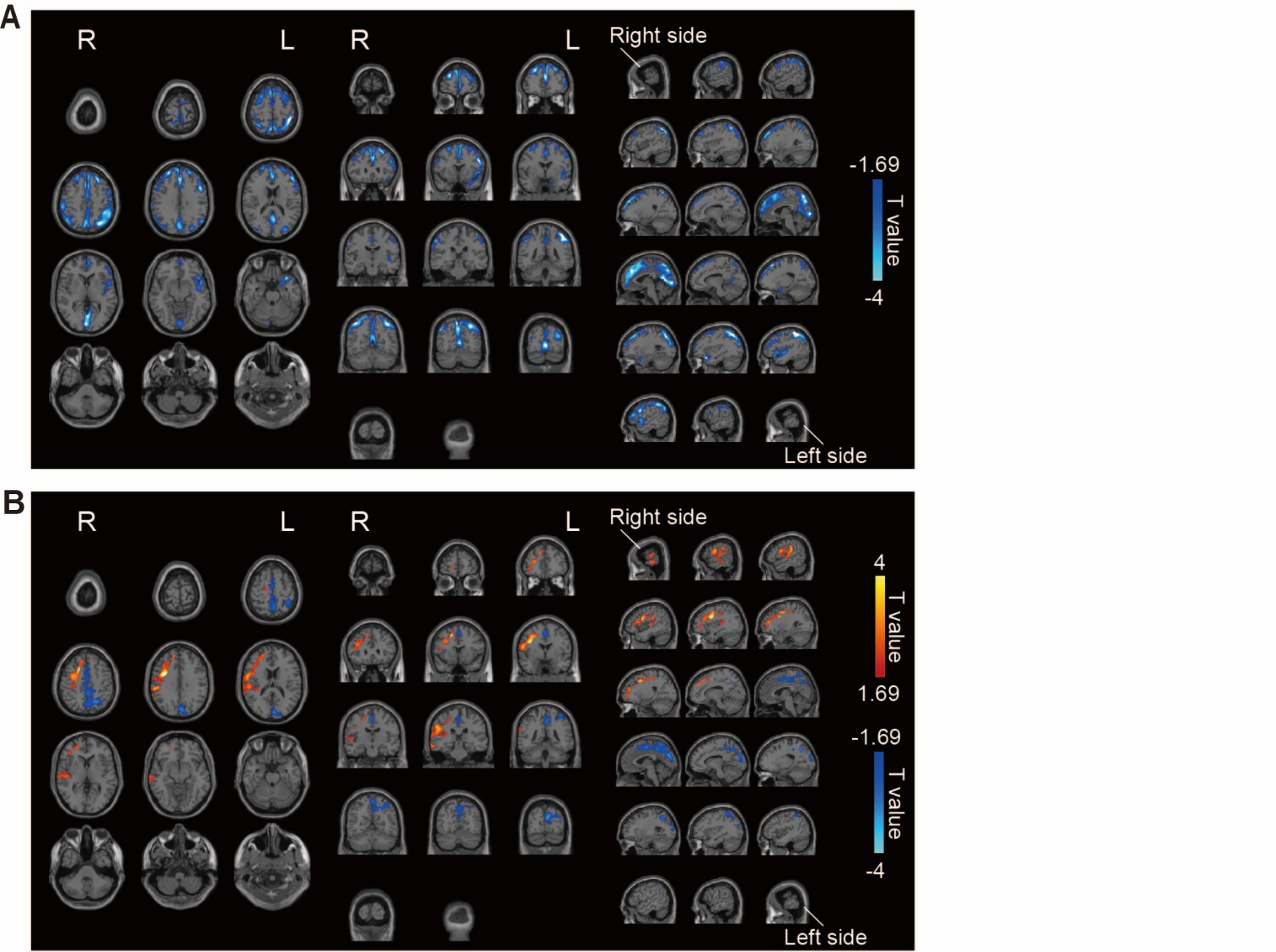
**

**Supplemental Fig. 5 Characterization of AMPAR distribution by age and sex in patients with TRD (brain wide presentation)**

**A,** Brain region showing a significant negative correlation between SUVR_30-50_ and age in patients with TRD (*p* < 0.05, negative correlation: *t* < -1.69, one-tailed, FDRc). **B,** Relative reduction (blue) and increase (red) of SUVR_30-50_ in male patients compared to female patients with TRD (*p* < 0.05, increase of SUVR_30-50_: *t* > 1.69, reduction of SUVR_30-50_: *t* < -1.69, one-tailed, FDRc). Significant clusters displayed on axial, coronal, sagittal slices. FDRc; false discovery rate correction.

**
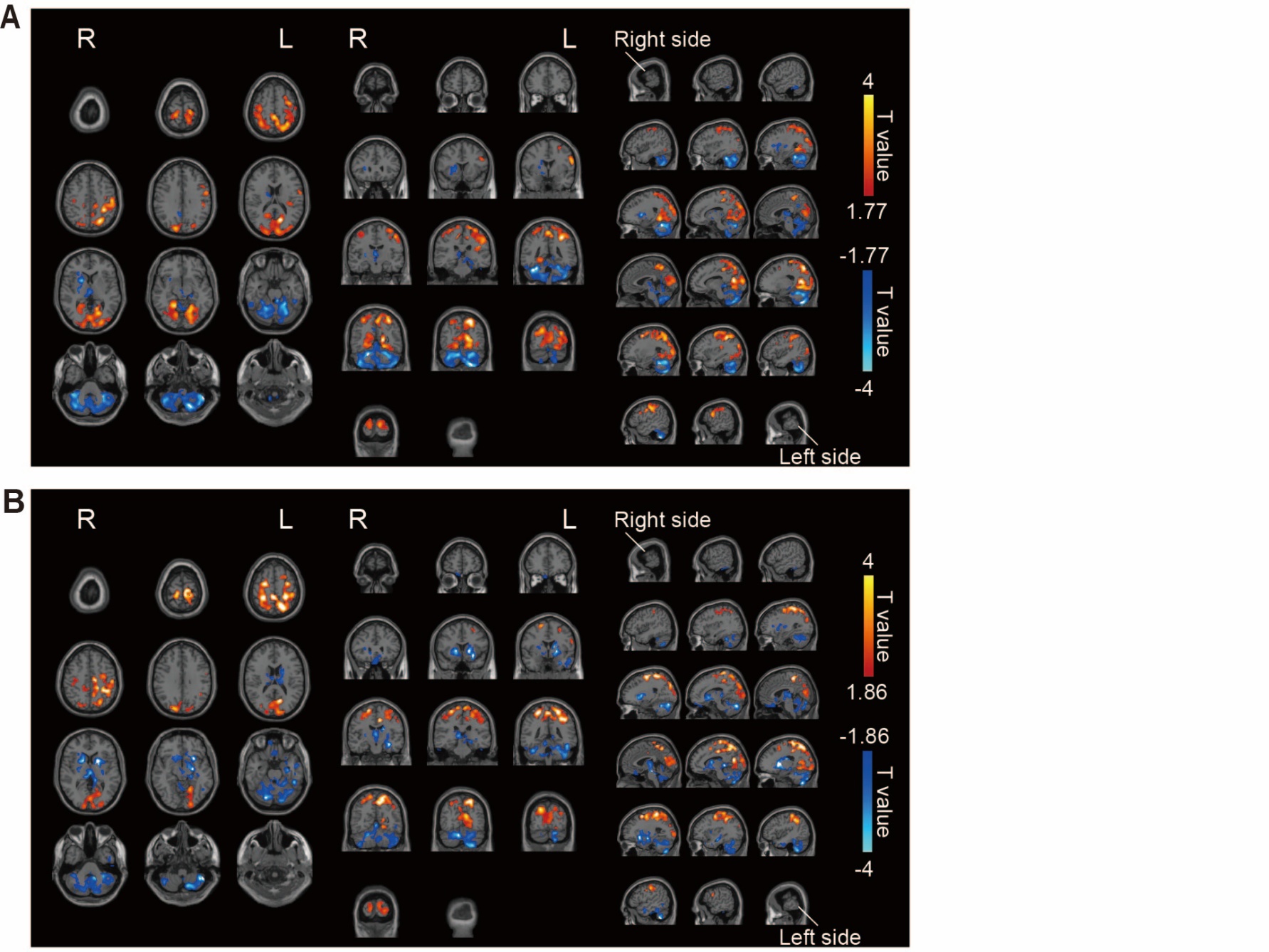
**

**Supplemental Fig. 6 Changes in cell surface AMPAR associated with symptom improvement induced by ketamine administration (brain wide presentation)**

**A, B,** Brain region showing a significant positive (red) and negative (blue) correlation between ΔSUVR_30-50_ and % change in MADRS in patients with TRD in the ketamine group (*p* < 0.05, positive correlation: *t* > 1.77 (**A**) and *t* > 1.86 (**B**), negative correlation: *t* < -1.77 (**A**) and *t* < -1.86 (**B**), one-tailed, FDRc). Significant clusters displayed on axial, coronal, sagittal slices**.** (**A**) This analysis was not adjusted for covariates. (**B**) This analysis was adjusted for covariates (age, sex, illness duration, number of failed medication trials and usage of benzodiazepines). FDRc; false discovery rate correction.

**
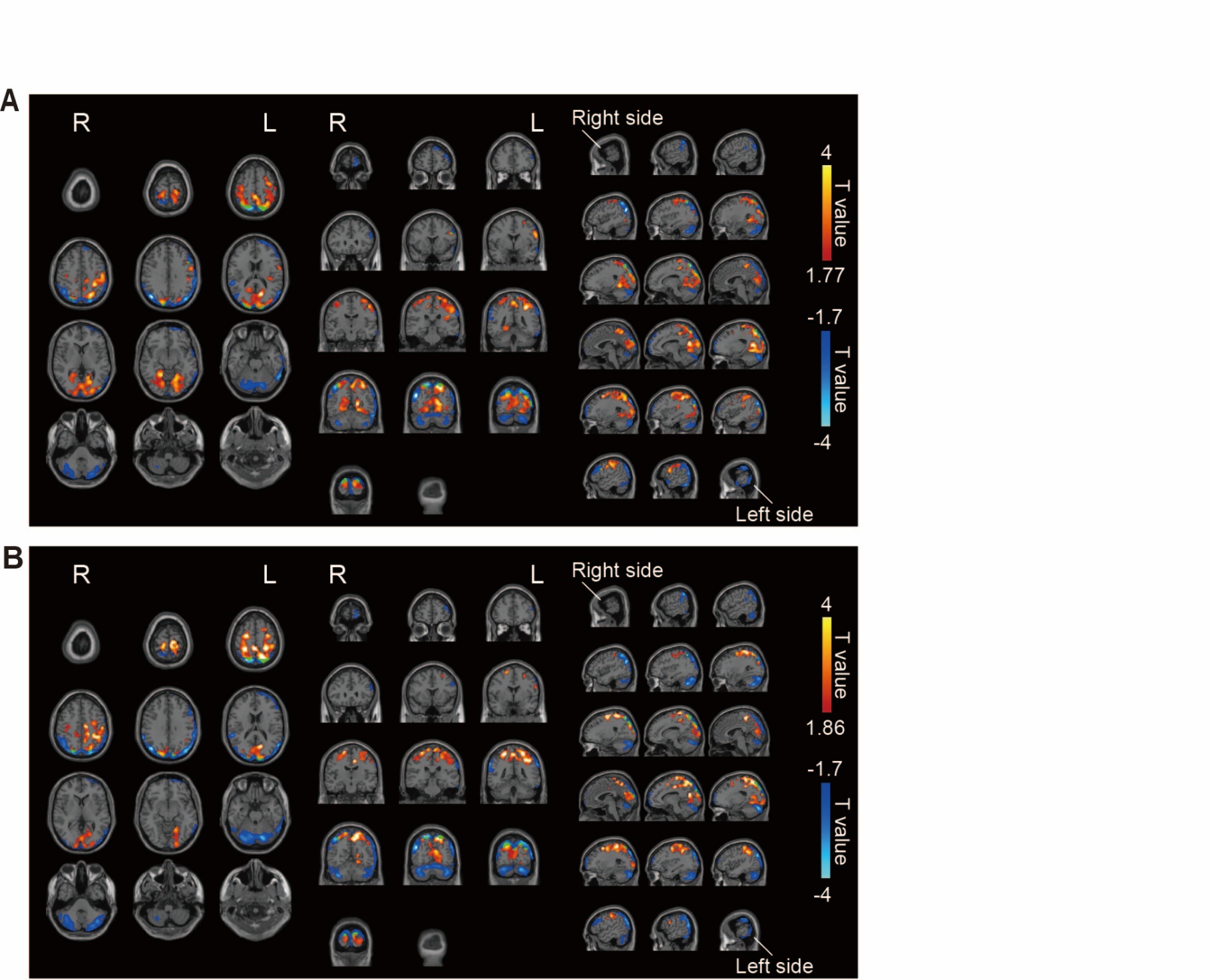
**

**Supplemental Fig. 7 Overlapping region where changes in AMPAR density correlate with clinical response to ketamine and region where AMPAR is altered in association with symptoms (brain wide presentation)**

**A, B,** Brain region showing a significant negative correlation between SUVR_30-50_ and the MADRS scores in patients with TRD (blue) (*p* < 0.05, *t* < -1.7, one-tailed, FDRc, adjusted covariate (age and sex (**A**); and age, sex, illness duration, number of failed medication trials and usage of benzodiazepines (**B**)) and a significant positive correlation between ΔSUVR_30-50_ and % change in MADRS in patients with TRD in the ketamine group (red) (*p* < 0.05, *t* > 1.77 (**A**) and *t* > 1.86 (**B**), one-tailed, FDRc, no adjusted covariate (**A**); and age, sex, illness duration, number of failed medication trials and usage of benzodiazepines (**B**)). Green region shows where the two regions overlap. Significant clusters and overlapping regions displayed on axial, coronal, sagittal slices. FDRc; false discovery rate correction.

**
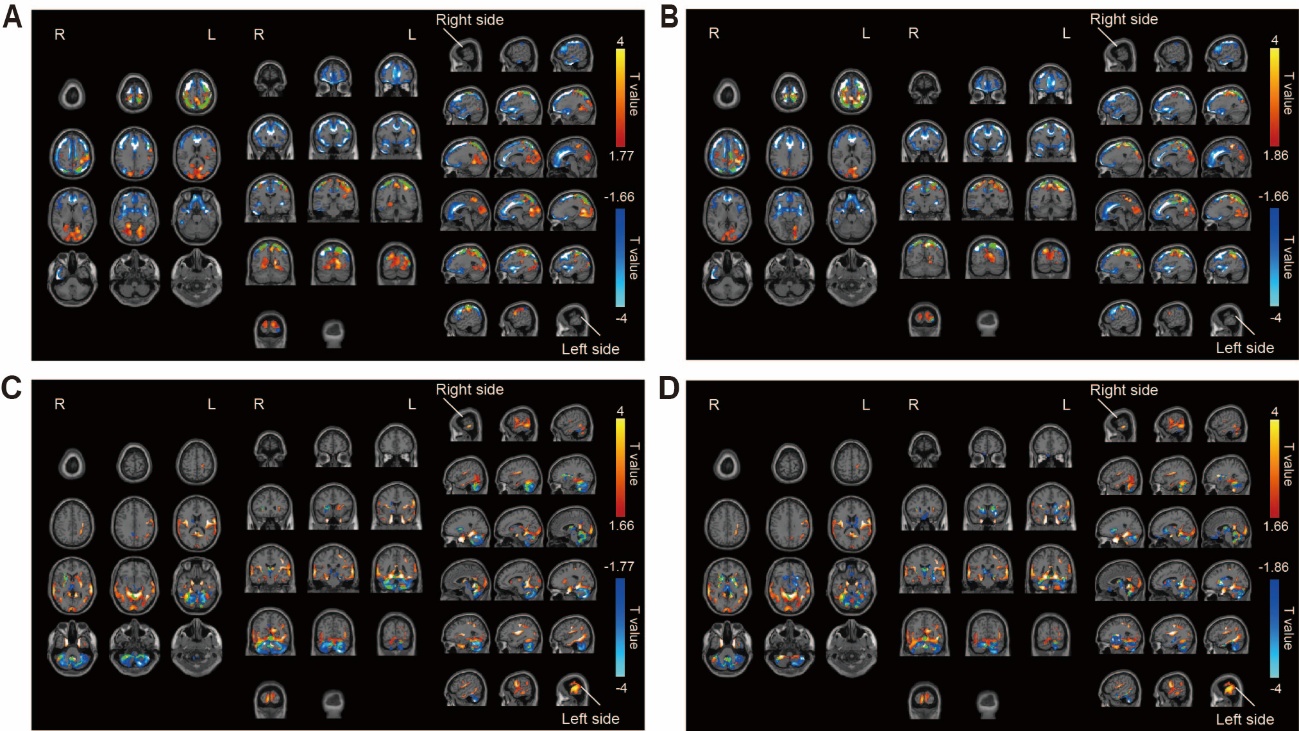
**

**Supplemental Fig. 8 Overlapping region where changes in AMPAR density correlate with clinical response to ketamine and region where AMPAR is different compared to healthy participants (brain wide presentation)**

**A, B,** Brain region showing a significant relative reduction of SUVR_30-50_ in patients with TRD compared to healthy participants (blue) (*p* < 0.05, *t* < -1.66, one-tailed, FDRc, adjusted covariate (age and sex) and a significant positive correlation between ΔSUVR_30-50_ and % change in MADRS in patients with TRD in the ketamine group (red) (*p* < 0.05, *t* > 1.77 (**A**) and *t* > 1.86 (**B**), one-tailed, FDRc, no adjusted covariate (**A**); and adjusted for covariates (age, sex, illness duration, number of failed medication trials and usage of benzodiazepines (**B**)). Green region shows where the two regions overlap. Significant clusters and overlapping regions displayed on an axial, coronal, sagittal slices. **C, D,** Brain regions showing a significant relative increase of SUVR_30-50_ in patients with TRD compared to healthy participants (red) (*p* < 0.05, *t* > 1.66, one-tailed, FDRc, adjusted covariate (age and sex) and a significant negative correlation between ΔSUVR_30-50_ and %change in MADRS in patients with TRD in the ketamine group (blue) (*p* < 0.05, *t* < -1.77 (**C**) and *t* < -1.86 (**D**), one-tailed, FDRc, no adjusted covariate (**C**); and adjusted for covariates (age, sex, illness duration, number of failed medication trials and usage of benzodiazepines (**D**)). Green region shows where the two regions overlap. Significant clusters and overlapping regions displayed on axial, coronal, sagittal slices. FDRc; false discovery rate correction.

**
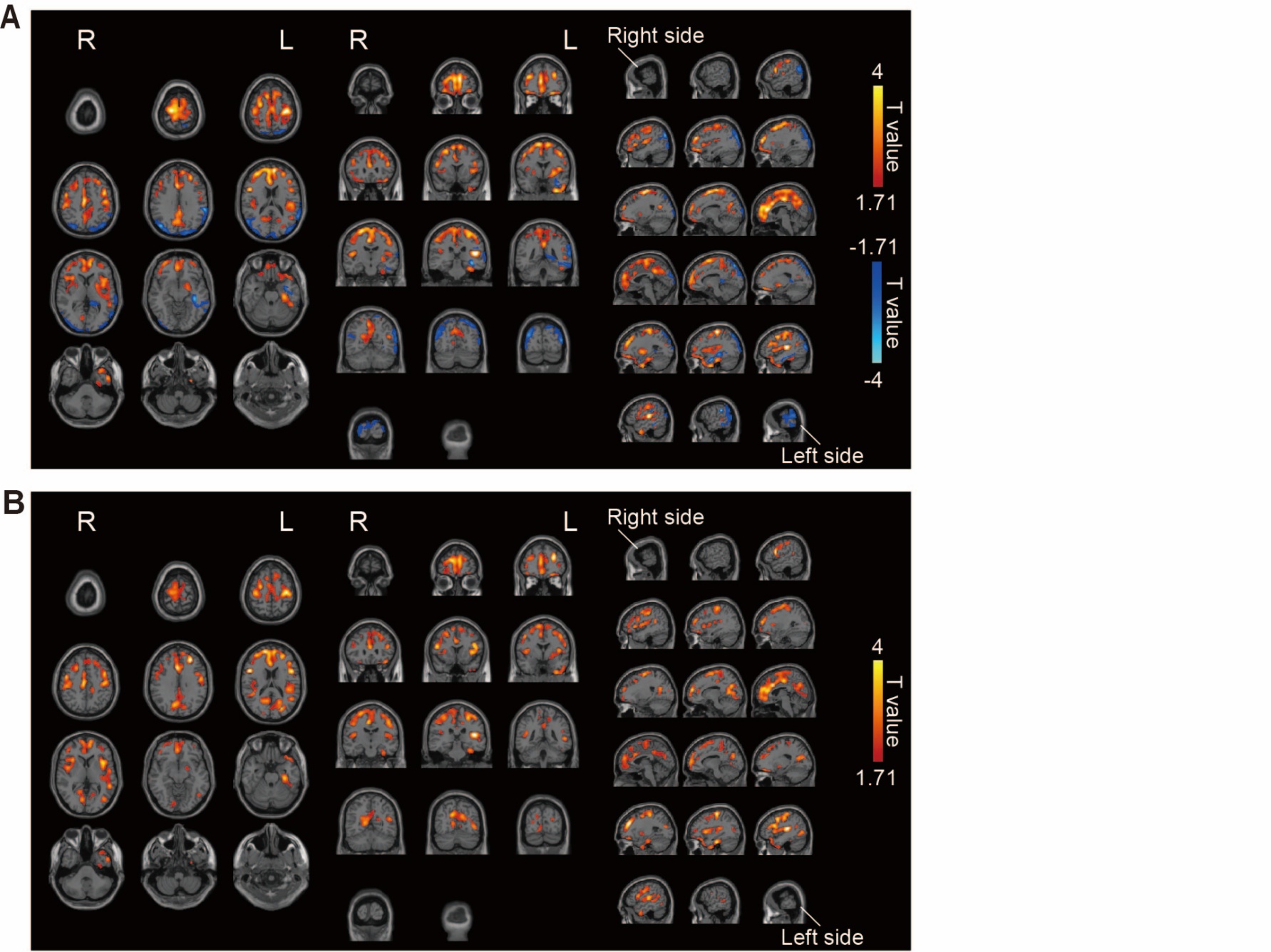
**

**Supplemental Fig. 9 Brain regions where AMPAR distribution predicts ketamine response in patients with TRD (brain wide presentation)**

**A,** Brain region showing a significant positive (red) and negative (blue) correlation between SUVR_30-50_ before ketamine administration and % change in MADRS (*p* < 0.05, positive correlation: *t* > 1.71, negative correlation: *t* < -1.71, one-tailed, FDRc, adjusted covariate (age and sex)). **B,** Brain region showing a significant positive correlation between SUVR_30-50_ before ketamine administration and % change in MADRS (adjusted covariates (age, sex, illness duration, number of failed medication trials and usage of benzodiazepines)). Significant clusters displayed on axial, coronal, sagittal slices. FDRc; false discovery rate correction.

**Supplemental Table 1. Demographic and Clinical Characteristics of Patients with TRD**

| No. | Age, years | Sex | DOI, years | MADRS  score | Medications prescribed, mg/day, Psychotropic drugs are written in italics |
| --- | --- | --- | --- | --- | --- |
| 1 | 49 | F | 28 | 28 | *Amoxapine 150, clomipramine 225, brexpiprazole 1, levomepromazine 45, clonazepam 2, methylphenidate 18, zolpidem 10, triazolam 0.5* |
| 2 | 37 | M | 20 | 23 | *Sertraline 75, clomipramine 30, lithium 800, clonazepam 0.5, zolpidem 5, suvorexant 20,* levothyroxine 1.5 (mcg) |
| 3 | 46 | F | 1.5 | 29 | *Venlafaxine 150, mirtazapine 15, aripiprazole 6* |
| 4 | 47 | M | 17 | 25 | *Nortriptyline 70, aripiprazole 6, lithium 400, lorazepam 0.5, zolpidem 10* |
| 5 | 33 | M | 7 | 27 | *Vortioxetine 20, venlafaxine 150, trazodone 25, brotizolam 0.25, flunitrazepam 1, suvorexant 20* |
| 6 | 46 | M | 1.5 | 22 | *Duloxetine 60, bromazepam 12, lithium 200* |
| 7 | 47 | M | 18 | 15 | *Venlafaxine 225, mirtazapine 30, aripiprazole 6, lorazepam 0.5* |
| 8 | 52 | M | 1 | 21 | *Sertraline 100, mirtazapine 30, trazodone 25* |
| 9 | 36 | F | 1.5 | 29 | *Escitalopram 20, mirtazapine 15, zolpidem 5, nitrazepam 10* |
| 10 | 59 | M | 3 | 25 | *Clomipramine 275, mirtazapine 30, aripiprazole 3, alprazolam 0.4, suvorexant 10* |
| 11 | 49 | M | 8 | 30 | *Selegiline 10, trazodone 50, lithium 600, flunitrazepam 1, zolpidem 10* |
| 12 | 49 | M | 4 | 28 | *Venlafaxine 225, mianserin 15, risperidone 1, ethyl loflazepate 1* |
| 13 | 30 | F | 6 | 37 | *Venlafaxine 225, aripiprazole 6, brotizolam 0.25, triazolam 0.25* |
| 14 | 29 | M | 3 | 33 | *Venlafaxine 225, aripiprazole 3, levomepromazine 10, suvorexant 20* |
| 15 | 26 | M | 10 | 23 | *Vortioxetine 20, clomipramine 150, olanzapine 5, lithium 600, eszopiclone 1* |
| 16 | 46 | M | 8 | 43 | *Clomipramine 180, mirtazapine 22.5, aripiprazole 3, nitrazepam 10, ramelteon 8* |
| 17 | 57 | M | 17 | 22 | *Amitriptyline 225, lormetazepam 1, eszopiclone 1* |
| 18 | 57 | M | 29 | 18 | *Escitalopram 20, duloxetine 40, aripiprazole 1, clonazepam 1.5, zolpidem 5, lemborexant 5* |
| 19 | 34 | F | 10 | 28 | *Amoxapine 75, escitalopram 5, chlorpromazine 12.5, lithium 400, alprazolam 1.2, flunitrazepam 2,* biperiden 6 |
| 20 | 32 | F | 12 | 34 | *Paroxetine 37.5, methylphenidate 63, clonazepam 0.5, bromazepam 2, topiramate 50* |
| 21 | 51 | M | 21 | 32 | *Vortioxetine 20, valproate 400, lurasidone 40, chlorpromazine 100, eszopiclone 3* |
| 22 | 37 | M | 7 | 43 | *Escitalopram 20, brexpiprazole 1, quetiapine fumarate 300* |
| 23 | 45 | M | 7 | 15 | *Nortriptyline 75, aripiprazole 0.5, clonazepam 1, ramelteon 8* |
| 24 | 31 | F | 15 | 16 | *Venlafaxine 37.5, brotizolam 0.25* |
| 25 | 37 | M | 4 | 40 | *Sertraline 100, paroxetine 37.5, lorazepam 1, triazolam 0.5, flunitrazepam 2* |
| 26 | 27 | M | 8 | 29 | *Duloxetine 40, dosulepin 25, ramelteon 8* |
| 27 | 41 | M | 24 | 36 | *Methlphenidate 72, flunitrazepam 2* |
| 28 | 33 | F | 3 | 22 | *Clomipramine 125, lorazepam 1* |
| 29 | 55 | M | 25 | 27 | *Nortriptyline 100, sulpiride 100* |
| 30 | 30 | F | 7 | 22 | *Venlafaxine 225, triazolam 0.25, aripiprazole 3* |
| 31 | 44 | F | 24 | 27 | *Lurasidone 20, lemborexant 10* |
| 32 | 39 | M | 19 | 28 | *Mirtazapine 15* |
| 33 | 31 | F | 10 | 44 | *Escitalopram 10, trazodone 100, valproate 400, lurasidone 60, bromazepam 6, etizolam 3, suvorexant 20, flunitrazepam 2* |
| 34 | 45 | M | 14 | 33 | *Escitalopram 20, aripiprazole 1, lemborexant 5, suvorexant 15, nitrazepam 5* |

M, male; F, female; DOI, duration of illness; MADRS, Montgomery–Åsberg Depression Rating Scale

**Supplemental Table 2. Changes in the MADRS total score among patients in the ketamine group during the double-blind period**

| No. | Baseline score | Post-treatment score | Score change  (% improvement in MADRS) |
| --- | --- | --- | --- |
| 1 | 28 | 22 | -6 (21.4 %) |
| 3 | 29 | 30 | 1 (-3.4 %) |
| 5 | 27 | 17 | -10 (37.0 %) |
| 8 | 21 | 16 | -5 (23.8 %) |
| 11 | 30 | 20 | -10 (33.3 %) |
| 12 | 28 | NA | NA |
| 13 | 37 | 32 | -5 (13.5 %) |
| 15 | 23 | 12 | -11 (47.8 %) |
| 18 | 18 | 20 | 2 (-11.1 %) |
| 20 | 34 | 28 | -6 (17.6 %) |
| 22 | 43 | 9 | -34 (79.1 %) |
| 23 | 15 | 2 | -13 (86.7 %) |
| 26 | 29 | 24 | -5 (17.2 %) |
| 27 | 36 | 25 | -11 (30.6 %) |
| 30 | 22 | 26 | 4 (-18.2 %) |
| 33 | 44 | NA | NA |
| 34 | 33 | 5 | -28 (84.8 %) |

MADRS, Montgomery–Åsberg Depression Rating Scale; NA, not applicable.

**Supplemental Table 3. Changes in the MADRS total score among patients in the placebo group during the double-blind and open-label periods**

| No. | Baseline score^†^ | Post-treatment score^†^ | Score change^†^  (% improvement in MADRS) | Baseline score^⁋^ | Post-treatment score^⁋^ | Score change^⁋^  (% improvement in MADRS) |
| --- | --- | --- | --- | --- | --- | --- |
| 2 | 23 | 26 | 3 (-13.0 %) | 34 | 25 | -9 (26.5 %) |
| 4 | 25 | 21 | -4 (16.0 %) | 22 | 15 | -7 (31.8 %) |
| 6 | 22 | 19 | -3 (13.6 %) | 36 | 29 | -7 (19.4 %) |
| 7 | 15 | 9 | -6 (40.0 %) | 16 | 0 | -16 (100 %) |
| 9 | 29 | 20 | -9 (31.0 %) | 23 | 23 | 0 (0 %) |
| 10 | 25 | 17 | -8 (32.0 %) | 20 | 9 | -11 (55.0 %) |
| 14 | 33 | 23 | -10 (30.3 %) | 24 | 16 | -8 (33.3 %) |
| 16 | 43 | 41 | -2 (4.7 %) | 43 | 8 | -35 (81.4 %) |
| 17 | 22 | 24 | 2 (-9.1 %) | 22 | 16 | -6 (27.3 %) |
| 19 | 28 | NA | NA | NA | NA | NA |
| 21 | 32 | 21 | -11 (34.4 %) | 21 | 15 | -6 (28.6 %) |
| 24 | 16 | 24 | 8 (-50.0 %) | 14 | NA | NA |
| 25 | 40 | 37 | -3 (7.5 %) | 42 | 18 | -24 (57.1 %) |
| 28 | 22 | 25 | 3 (-13.6 %) | 24 | 16 | -8 (33.3 %) |
| 29 | 27 | 25 | -2 (7.4 %) | 28 | 20 | -8 (28.6 %) |
| 31 | 27 | 25 | -2 (7.4 %) | 27 | 8 | -19 (70.4 %) |
| 32 | 28 | 29 | 1 (-3.6%) | 35 | 32 | -3 (8.6 %) |

^†^Double-blind period.

^⁋^Open-label period.

MADRS, Montgomery–Åsberg Depression Rating Scale; NA, not applicable.

**Supplemental Table 4. Brain regions showing a significant negative correlation between SUVR_30-50_ and the MADRS scores in patients with TRD (based on Fig. 1A and Supplemental Fig. 4A)**

| Brain regions | | R/L |
| --- | --- | --- |
| Category | Detail |  |
| Frontal lobe | Superior frontal gyrus | L |
|  | Middle frontal gyrus | L |
|  | Inferior frontal gyrus | L |
|  | Precentral gyrus | L |
| Temporal lobe | Superior temporal gyrus (posterior part) | R |
|  | Middle temporal gyrus (anterior part) | R/L |
|  | Inferior temporal gyrus (lateral part) | L |
|  | Angular gyrus | R/L |
| Parietal lobe | Superior parietal gyrus | R/L |
|  | Inferior parietal gyrus | R/L |
|  | Precuneus | R/L |
|  | Supramarginal gyrus (lateral part) | R/L |
| Occipital lobe | Superior occipital gyrus | R/L |
|  | Middle occipital gyrus | R/L |
|  | Cuneus | R/L |
| Cerebellum | Cortex | R/L |

Abbreviations: R, Right; L, Left.

**Supplemental Table 5. Brain regions showing a significant difference in AMPAR density between healthy participants and patients with TRD (based on Fig. 1B and Supplemental Fig. 4C)**

| Brain regions which decreased AMPAR density in TRD | | | Brain regions which increased AMPAR density in TRD | | |
| --- | --- | --- | --- | --- | --- |
| Brain regions | | R/L | Brain regions | | R/L |
| Category | Detail |  | Category | Detail |  |
| Frontal lobe | Superior frontal gyrus | R/L | Temporal lobe | Superior temporal gyrus (anterior part) | R/L |
|  | Middle frontal gyrus | R/L |  | Middle temporal gyrus (posterior part) | R/L |
|  | Inferior frontal gyrus | R/L |  | Inferior temporal gyrus (medial part) | R/L |
|  | Orbitofrontal gyrus | R/L |  | Temporal pole (medial part) | R/L |
|  | Supplementary motor area | R/L |  | Hippocampus | R/L |
|  | Precentral gyrus | R/L |  | Parahippocampal gyrus | R/L |
| Temporal lobe | Temporal pole (lateral part) | R |  | Fusiform gyrus | R/L |
|  | Amygdala | R/L | Parietal lobe | Supramarginal gyrus (partially ventral part) | R/L |
|  | Angular gyrus | R/L | Occipital lobe | Calcarine | R/L |
| Parietal lobe | Superior parietal gyrus | R/L |  | Lingual gyrus | R/L |
|  | Inferior parietal gyrus | R/L | Insula | Posterior part | R/L |
|  | Precuneus | R/L | Thalamus |  | R/L |
|  | Postcentral gyrus | R/L | Basal ganglia | Caudate | R/L |
| Occipital lobe | Superior occipital gyrus | R/L |  | Putamen | R/L |
|  | Middle occipital gyrus | R/L |  | Pallidum | R/L |
|  | Inferior occipital gyrus | R | Cerebellum | Vermis, Cortex | R/L |
| Cingulate cortex | Anterior cingulate gyrus | R/L |  |  |  |
|  | Middle cingulate gyrus | R/L |  |  |  |
| Insula | Anterior part | R/L |  |  |  |

Abbreviations: R, Right; L, Left.

**Supplemental Table 6. Brain regions showing a significant negative correlation between AMPAR density and age (based on Supplemental Fig. 5A)**

| Brain regions | | R/L |
| --- | --- | --- |
| Category | Detail |  |
| Frontal lobe | Superior frontal gyrus | R/L |
|  | Middle frontal gyrus | R/L |
|  | Inferior frontal gyrus | R/L |
|  | Precentral gyrus | R/L |
|  | Supplementary motor area | R/L |
| Temporal lobe | Superior parietal gyrus | L |
|  | Parahippocampal gyrus | L |
| Parietal lobe | Superior parietal gyrus | R/L |
|  | Inferior parietal gyrus | R/L |
|  | Postcentral gyrus | R/L |
|  | Precuneus | R/L |
| Occipital lobe | Middle occipital gyrus | R/L |
|  | Cuneus | R/L |
|  | Lingual | R/L |
|  | Calcarine | R/L |
| Cingulate cortex | Anterior cingulate gyrus | R/L |
|  | Middle cingulate gyrus | R/L |
|  | Post cingulate gyrus | R/L |
| Insula |  | L |

Abbreviations: R, Right; L, Left.

**Supplemental Table 7. Brain regions showing a significant difference in AMPAR density between male and female patients with TRD (based on Supplemental Fig. 5B)**

| Brain regions which increased AMPAR density in male patients | | | Brain regions which decreased AMPAR density in male patients | | |
| --- | --- | --- | --- | --- | --- |
| Brain area | | R/L | Brain area | | R/L |
| Category | Detail |  | Category | Detail |  |
| Frontal lobe | Superior frontal gyrus | R | Frontal lobe | Supplementary motor area | R/L |
|  | Middle frontal gyrus | R | Parietal lobe | Precuneus | R/L |
|  | Inferior frontal gyrus | R | Occipital lobe | Cuneus | R/L |
|  | Precentral gyrus | R |  | Calcarine | R/L |
| Temporal lobe | Superior parietal gyrus | R |  | Superior occipital gyrus | L |
|  | Middle temporal gyrus | R | Cingulate cortex | Middle cingulate gyrus | R/L |
| Parietal lobe | Supramarginal gyrus | R |  |  |  |
|  | Postcentral gyrus | R |  |  |  |

Abbreviations: R, Right; L, Left.

**Supplemental Table 8. Brain regions showing a significant correlation between change in AMPAR density and symptom improvement induced by ketamine administration (based on Fig. 2A and Supplemental Fig. 6A)**

| Positively correlated regions | | | Negatively correlated regions | | |
| --- | --- | --- | --- | --- | --- |
| Brain regions | | R/L | Brain regions | | R/L |
| Category | Detail |  | Category | Detail |  |
| Frontal lobe | Superior frontal gyrus | L | Temporal lobe | Parahippocampal gyrus | L |
|  | Middle frontal gyrus | L | Thalamus |  | R/L |
|  | Inferior frontal gyrus | L | Basal ganglia | Caudate | R |
|  | Precentral gyrus | R/L |  | Putamen | R |
| Parietal lobe | Superior parietal gyrus | R/L |  | Pallidum | R |
|  | Inferior parietal gyrus | R/L | Habenula |  | R |
|  | Precuneus | R/L | Cerebellum | Vermis, Cortex | R/L |
|  | Postcentral gyrus | R/L |  |  |  |
| Occipital lobe | Middle occipital gyrus | R/L |  |  |  |
|  | Inferior occipital gyrus | R/L |  |  |  |
|  | Cuneus | R/L |  |  |  |
|  | Calcarine | R/L |  |  |  |
|  | Lingual gyrus | R/L |  |  |  |
| Cingulate cortex | Middle cingulate gyrus | R/L |  |  |  |

Abbreviations: R, Right; L, Left.

**Supplemental Table 9. Overlapping regions where changes in AMPAR density correlate with clinical response to ketamine and regions where AMPAR density is altered in association with symptoms (based on Fig. 3 and Supplemental Fig. 7A)**

| Brain regions | | R/L |
| --- | --- | --- |
| Category | Detail |  |
| Frontal lobe | Middle frontal gyrus | L |
|  | Precentral gyrus | L |
| Parietal lobe | Superior parietal gyrus | R/L |
|  | Precuneus | L |
| Occipital lobe | Superior occipital gyrus | R/L |
|  | Middle occipital gyrus | R/L |
|  | Cuneus | R/L |

Abbreviations: R, Right; L, Left.

**Supplemental Table 10. Overlapping regions where changes in AMPAR density correlate with clinical response to ketamine and regions where AMPAR density is different compared to healthy participants (based on Fig. 4 and Supplemental Fig. 8A, 8C)**

| Overlapping area based on Fig. 4A and Supplemental Fig. 8A | | | Overlapping area based on Fig. 4C and Supplemental Fig. 8C | | |
| --- | --- | --- | --- | --- | --- |
| Brain regions | | R/L | Brain regions | | R/L |
| Category | Detail |  | Category | Detail |  |
| Frontal lobe | Superior frontal gyrus | L | Temporal lobe | Parahippocampal gyrus | L |
|  | Middle frontal gyrus | L | Thalamus |  | R |
|  | Inferior frontal gyrus | L | Basal ganglia | Caudate | R |
|  | Precentral gyrus | R/L |  | Putamen | R |
| Parietal lobe | Superior parietal gyrus | R/L |  | Pallidum | R |
|  | Inferior parietal gyrus | R/L | Cerebellum | Vermis, cortex | R/L |
|  | Precuneus | R/L |  |  |  |
|  | Postcentral gyrus | R/L |  |  |  |
| Occipital lobe | Middle occipital gyrus | R/L |  |  |  |
|  | Inferior occipital gyrus | R/L |  |  |  |
|  | Cuneus | R/L |  |  |  |
| Cingulate cortex | Middle cingulate gyrus | R/L |  |  |  |

Abbreviations: R, Right; L, Left.

**Supplemental Table 11. Brain regions where AMPAR distribution predicts ketamine response in patients with TRD (based on Fig. 5 and Supplemental Fig. 9A)**

| Positively correlated regions | | | Negatively correlated regions | | |
| --- | --- | --- | --- | --- | --- |
| Brain regions | | R/L | Brain regions | | R/L |
| Category | Detail |  | Category | Detail |  |
| Frontal lobe | Superior frontal gyrus | R/L | Temporal lobe | Superior temporal gyrus | L |
|  | Middle frontal gyrus | R/L |  | Middle temporal gyrus | L |
|  | Inferior frontal gyrus | R/L |  | Inferior temporal gyrus | L |
|  | Orbitofrontal gyrus | R/L |  | Hippocampus | L |
|  | Supplementary motor area | R/L |  | Amygdala | L |
|  | Precentral gyrus | R/L |  | Fusiform gyrus | L |
| Parietal lobe | Superior parietal gyrus | R/L |  | Angular gyrus | R/L |
|  | Inferior parietal gyrus | R/L | Parietal lobe | Inferior parietal gyrus | R/L |
|  | Precuneus (medial part) | R/L |  | Supramarginal gyrus | R/L |
|  | Postcentral gyrus | R/L |  | Precuneus (lateral part) | R/L |
| Temporal lobe | Temporal pole | L | Occipital lobe | Cuneus | R/L |
|  | Superior temporal gyrus | R/L |  | Calcarine | R |
|  | Middle temporal gyrus | L |  |  |  |
|  | Inferior temporal gyrus | L |  |  |  |
|  | Fusiform gyrus | L |  |  |  |
| Occipital lobe | Cuneus | R/L |  |  |  |
|  | Calcarine | R |  |  |  |
| Cingulate cortex | Anterior cingulate gyrus | R/L |  |  |  |
|  | Middle cingulate gyrus | R/L |  |  |  |
|  | Posterior cingulate gyrus | R/L |  |  |  |
| Insula |  | R/L |  |  |  |
| Basal ganglia | Putamen | R/L |  |  |  |
|  | Pallidum | R/L |  |  |  |

Abbreviations: R, Right; L, Left.
